# Global Human Footprint on the Linkage between Biodiversity and Ecosystem Functioning in Reef Fishes

**DOI:** 10.1371/journal.pbio.1000606

**Published:** 2011-04-05

**Authors:** Camilo Mora, Octavio Aburto-Oropeza, Arturo Ayala Bocos, Paula M. Ayotte, Stuart Banks, Andrew G. Bauman, Maria Beger, Sandra Bessudo, David J. Booth, Eran Brokovich, Andrew Brooks, Pascale Chabanet, Joshua E. Cinner, Jorge Cortés, Juan J. Cruz-Motta, Amilcar Cupul Magaña, Edward E. DeMartini, Graham J. Edgar, David A. Feary, Sebastian C. A. Ferse, Alan M. Friedlander, Kevin J. Gaston, Charlotte Gough, Nicholas A. J. Graham, Alison Green, Hector Guzman, Marah Hardt, Michel Kulbicki, Yves Letourneur, Andres López Pérez, Michel Loreau, Yossi Loya, Camilo Martinez, Ismael Mascareñas-Osorio, Tau Morove, Marc-Olivier Nadon, Yohei Nakamura, Gustavo Paredes, Nicholas V. C. Polunin, Morgan S. Pratchett, Héctor Reyes Bonilla, Fernando Rivera, Enric Sala, Stuart A. Sandin, German Soler, Rick Stuart-Smith, Emmanuel Tessier, Derek P. Tittensor, Mark Tupper, Paolo Usseglio, Laurent Vigliola, Laurent Wantiez, Ivor Williams, Shaun K. Wilson, Fernando A. Zapata

**Affiliations:** 1 Department of Biology, Dalhousie University, Halifax, Nova Scotia, Canada; 2 Center for Marine Biodiversity and Conservation, Scripps Institution of Oceanography, La Jolla, California, United States of America; 3 Departamento de Biologia Marina, Universidad Autónoma de Baja California Sur, La Paz, México; 4 University of Hawaii Joint Institute for Marine and Atmospheric Research, Honolulu, Hawaii, United States of America; 5 Coral Reef Ecosystem Division, National Oceanic and Atmospheric Administration Pacific Islands Fisheries Science Center, Honolulu, Hawaii, United States of America; 6 Marine Research and Conservation, Charles Darwin Foundation, Galapagos Islands, Ecuador; 7 Institute for Water, Environment and Health, United Nations University, Hamilton, Ontario, Canada; 8 Australian Research Council Centre of Excellence for Coral Reef Studies, James Cook University, Townsville, Queensland, Australia; 9 Commonwealth Centre for Applied Environmental Decision Analysis, The School of Biological Sciences, University of Queensland, Brisbane, Queensland, Australia; 10 Fundación Malpelo y Otros Ecosistemas Marinos, Parques Nacionales Naturales de Colombia, Bogota, Colombia; 11 School of the Environment, University of Technology, Sydney, New South Wales, Australia; 12 Department of Zoology, Tel-Aviv University, Tel-Aviv, Israel; 13 Coastal Research Center, Marine Science Institute, University of California, Santa Barbara, California, United States of America; 14 Institut de Recherche pour le Développement, Reunion Island, France; 15 Centro de Investigación en Ciencias del Mar y Limnología, Universidad de Costa Rica, San José, Costa Rica; 16 Departamento de Estudios Ambientales, Universidad Simón Bolívar, Caracas, Venezuela; 17 Departamento de Ciencias Biológicas, Centro Universitario de la Costa, Universidad de Guadalajara, Puerto Vallarta, México; 18 Fisheries Research and Monitoring Division, Pacific Islands Fisheries Science Center, National Oceanic and Atmospheric Administration, Honolulu, Hawaii, United States of America; 19 Hawaii Institute of Marine Biology, University of Hawaii, Kaneohe, Hawaii, United States of America; 20 Institute of Marine and Antarctic Studies, University of Tasmania, Tasmania, Australia; 21 Leibniz Center for Tropical Marine Ecology, Bremen, Germany; 22 Department of Zoology, University of Hawaii, Honolulu, Hawaii, United States of America; 23 Biodiversity and Macroecology Group, Department of Animal and Plant Sciences, University of Sheffield, Sheffield, United Kingdom; 24 Blue Ventures, London, United Kingdom; 25 The Nature Conservancy, South Brisbane, Queensland, Australia; 26 Smithsonian Tropical Research Institute, Panama City, Panama; 27 Institut de Recherche pour le Développement, c/o Université de Perpignan, Perpignan, France; 28 Laboratoire Insulaire du Vivant et de L'environnement, Université de la Nouvelle-Calédonie, Nouméa Cedex, New Caledonia; 29 Laboratoire d'Ecologie Marine, Université de La Réunion, Saint-Denis Messageries, France; 30 Instituto de Recursos, Universidad del Mar, Oaxaca, Mexico; 31 Department of Biology, McGill University, Montreal, Quebec, Canada; 32 Secretaría Nacional de Planificación y Desarrollo, Quito, Ecuador; 33 Centro de Investigaciones Biológicas del Noroeste, Universidad Autónoma de Baja California Sur, La Paz, Mexico; 34 Papua New Guinea Marine Program, Wildlife Conservation Society, Kavieng, Papua New Guinea; 35 Graduate School of Kuroshio Science, Kochi University, Kochi, Japan; 36 Centro para la Biodiversidad Marina y Conservación del Golfo de California, San Diego, California, United States of America; 37 Marine Science and Technology, Newcastle University, Newcastle, United Kingdom; 38 Instituto Nazca de Investigaciones Marinas, Quito, Ecuador; 39 Centre d'Estudis Avançats de Blanes, Blanes, Spain; 40 National Geographic Society, Washington, District of Columbia, United States of America; 41 Réserve Naturelle Marine de La Réunion, Reunion Island, France; 42 United Nations Environment Programme World Conservation Monitoring Centre, Cambridge, United Kingdom; 43 Microsoft Research Computational Science Laboratory, Cambridge, United Kingdom; 44 The WorldFish Center, Penang, Malaysia; 45 Institut de Recherche pour le Développement, Nouméa Cedex, New Caledonia; 46 Aquarium des Lagons, Nouméa Cedex, New Caledonia; 47 Marine Science Program, Department of Environment and Conservation, Kensington, Western Australia, Australia; 48 Departamento de Biología, Universidad del Valle, Cali, Colombia; Cornell University, United States of America

## Abstract

Difficulties in scaling up theoretical and experimental results have raised controversy over the consequences of biodiversity loss for the functioning of natural ecosystems. Using a global survey of reef fish assemblages, we show that in contrast to previous theoretical and experimental studies, ecosystem functioning (as measured by standing biomass) scales in a non-saturating manner with biodiversity (as measured by species and functional richness) in this ecosystem. Our field study also shows a significant and negative interaction between human population density and biodiversity on ecosystem functioning (i.e., for the same human density there were larger reductions in standing biomass at more diverse reefs). Human effects were found to be related to fishing, coastal development, and land use stressors, and currently affect over 75% of the world's coral reefs. Our results indicate that the consequences of biodiversity loss in coral reefs have been considerably underestimated based on existing knowledge and that reef fish assemblages, particularly the most diverse, are greatly vulnerable to the expansion and intensity of anthropogenic stressors in coastal areas.

## Abstract 2

A global survey of reef fishes shows that the consequences of biodiversity loss are greater than previously anticipated as ecosystem functioning remained unsaturated with the addition of new species. Additionally, reefs worldwide, particularly those most diverse, are highly vulnerable to human impacts that are widespread and likely to worsen due to ongoing coastal overpopulation.

## Author Summary

The increasing intensity of human disturbance worldwide is triggering unprecedented biodiversity losses, which is raising concerns over whether ecosystems will work and continue delivering goods and services to humanity. In contrast to previous experimental studies, which describe saturating relationships between ecosystem functioning and biodiversity, we show that in reef fish systems, functioning (as standing biomass) accelerates with the addition of new species. This non-saturating relationship implies unique contributions of species to the functioning of this ecosystem and indicates that the consequences of losing biodiversity are significantly greater than previously anticipated. We also demonstrate a negative effect of human density on reef fish functioning such that for the same number of people the loss of standing biomass is significantly larger in more diverse ecosystems. Unfortunately, human effects can arise through multiple stressors (such as fishing, coastal development, and land use) and are widespread and likely to worsen, as some 75% of the world's coral reefs are currently nearby human settlements and because almost all countries with coral reefs are expected to double their populations within the next 50 to 100 years. Our results call for both further investigation of the impact of biodiversity on ecosystem functioning and strategies to manage and prevent the increasing intensity and expansion of anthropogenic stressors in coastal areas.

## Introduction

The growth and spatial expansion of the world's human population have inevitably been accompanied by changes in land use, pollution, and exploitation of natural resources [1], which in turn have raised concerns over the loss of species [2] and imperilment of ecosystem functioning [3]–[10]. Although theoretical and experimental studies have demonstrated that biodiversity loss is often detrimental to multiple ecosystem properties [4]–[8], the extrapolation of this finding to actual scenarios of human impacts remains contentious because of the difficulty in simulating the full complexity of natural ecosystems [7]–[10]. Thébault and Loreau [11], for instance, created a theoretical model and concluded that ecosystem functioning “does not always increase with…diversity and that changes in biodiversity can lead to complex if predictable changes in ecosystem processes.” Similarly, Fukami and Morin [12] showed that the history of colonization can yield different forms of the biodiversity–ecosystem functioning relationship. Finally, Cardinale et al. [13] demonstrated the theoretical role of scale, disturbances, and dispersal in shaping biodiversity–functioning relationships. As noted, theoretical and experimental approaches have also generated a case for reasonable doubt about the extrapolation of results to natural conditions, and this has been outlined in several recent meta-analyses. For instance, Hillebrand and Matthiessen [9] concluded that “empirical and theoretical studies do not reflect the complexity of natural ecosystems, which makes it difficult to transfer the results to natural situations of species loss.” Likewise, Balvanera et al. [7] stated that “simple generalizations among ecosystem types, ecosystems properties or trophic level manipulated or measured will be difficult to sustain.” Similarly, Srivastava and Vellend [8] argued that “although there is substantial evidence that biodiversity is able to affect function, particularly for plant communities, it is unclear if these patterns will hold for realistic scenarios of extinction, multitrophic communities, or larger scales.” Finally, Duffy [10], who reviewed the emerging issue of the scalability of small-scale experiments to the real world, concluded that previous research may have greatly underestimated the real effects of diversity on the functioning of natural ecosystems. In short, theoretical and experimental studies have provided a fascinating view into the ecosystem consequences of biodiversity loss, but these same studies also show that there is uncertainty in the extrapolation of their results to natural conditions. Unfortunately, assessment of the relationship between biodiversity and functioning in natural ecosystems remains rare, particularly at landscape and regional levels [4],[6],[7]. The limited validation of experimental and theoretical studies, combined with the increasing expansion and intensity of human stressors [1], highlights a key research gap that has been referred as a “major future challenge” of modern ecology [4].

**Figure 1:**
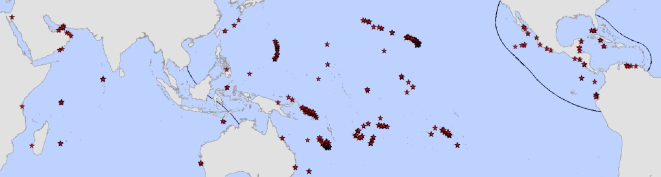
Sampled locations. Red stars represent sample locations. Regions analyzed are separated with solid black lines.

In this study, we carried out a global survey of reef fish assemblages (Figure 1; Tables S1 and S2) to assess the link between diversity and functioning in a natural ecosystem. Our study provides a real and relevant framework for evaluating the role of biodiversity on ecosystem functioning. However, in contrast to experimental and theoretical studies where alternative drivers can be controlled, field studies like ours can be confounded by the effects of additional variables that are impossible to manipulate under natural conditions. To address this issue, we assessed the relationship between biodiversity and ecosystem functioning while simultaneously examining the role of alternative factors including environmental, physiographic, and anthropogenic variables (see Materials and Methods). We assessed these relationships with the use of structural equation modeling, a statistical framework that evaluates the causality of relationships in the face of collinear and/or confounding variables [14]. In contrast to previous experimental and theoretical work, our study of a natural ecosystem demonstrates that the relationship between biodiversity and function is non-saturating (this pattern was consistent among regions and robust to the effects of several confounding factors; Figures 1 and S1, S2, S3; Table S3). We also found a negative interaction between human density and biodiversity such that the deleterious effects of human density on standing biomass were stronger at more diverse reefs. The existence of a non-saturating relationship between standing biomass and biodiversity, at least for coral reefs, indicates that the consequences of losing biodiversity may be significantly greater than previously anticipated, while the reduction of biomass due to human density suggests that all reefs, particularly those that are more diverse, are highly vulnerable to the expansion and increasing intensity of human activities in coastal areas.

## Results/Discussion

Here we used the standing biomass of reef fish assemblages as our metric of ecosystem functioning. This metric has been one of the primary proxies of ecosystem functioning used in previous experimental studies [5],[15] and is directly and/or indirectly relevant to the full extent of properties implied in the definition of ecosystem functioning. In a broad sense, ecosystem functioning is a term used to encompass a variety of ecosystem properties related to “pools” and “fluxes” of matter and energy [4] and the ecosystem provision of goods and services [6],[16]. Standing biomass is already a “pool” of matter, and through food provision it represents one of the main ecosystem services that coral reefs provide to human societies [17]. There is a strong relationship between body mass and metabolic energy requirements in fishes (e.g., [18],[19]), and thus an accurate surrogacy between standing biomass and energy fluxes in fish assemblages (e.g., [19],[20]; see also demonstration in Figure S1, Text S1, and Tables S4 and S5).

**Figure 2:**
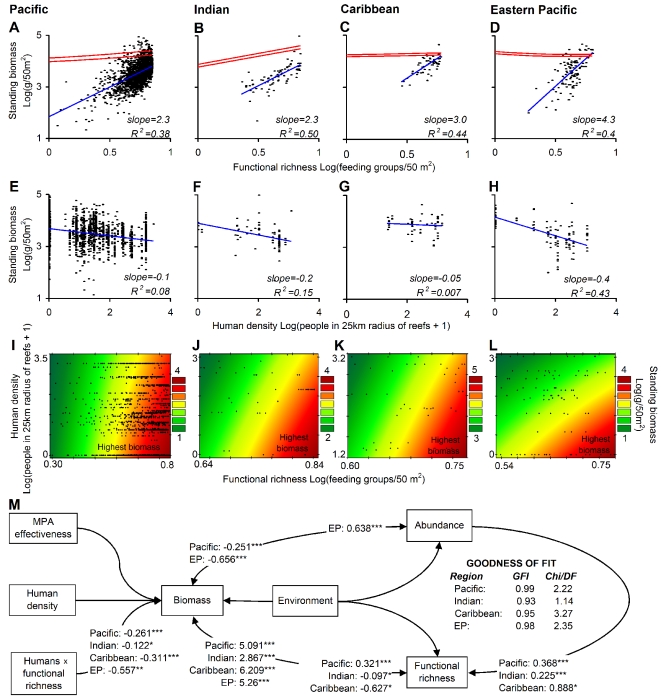
Predictors of standing biomass in reef fishes. (A–D) Plots showing the relationship between standing biomass and functional richness for each region. Plots are on a logarithmic scale because this produces better fit to the data (Table S3; see Figure S2 for plots with species richness and Figure S3 for plots controlling for abundance of individuals). Blue lines indicate the linear trend fitted to the data, while red lines indicate 95% confidence intervals around the mean trend line relating standing biomass and diversity as calculated from the null model described in the text (results based on 100 runs of such null model). (E–H) Plots depicting the relationship between standing biomass and human population density. (I–L) Plots outlining changes in standing biomass as calculated from estimates of its covariance with functional richness and human population density as predicted from the structural equation model shown in (M). Fitting the equation that predicts standing biomass from human population density and functional richness is superior to fitting a trend surface over the raw data, as the former accounts for other variables (Figure S5 shows fits to the raw data). Equations were fitted only over the range of values of the data collected, which are indicated with black dots. (M) Diagram showing the unstandardized covariance estimates for the relationships in the structural equation model run independently for each region. All variables were log-transformed. The goodness-of-fit metrics are shown inside (M). The best model for each region included the variables for which the unstandardized covariance estimates are shown. Statistical significance for all relationships is best assessed from the results of the structural equation model given the control of confounding factors. Significance of covariance estimates with critical ratios significant at *p*<0.0001 (***), *p*<0.001 (**), and *p*<0.01 (*) are indicated beside each estimate. Chi/DF, Chi-square divided by the degrees of freedom; EP, Eastern Pacific; GFI, goodness-of-fit index.

In previous experimental studies, the relationship between ecosystem functioning and biodiversity has yielded saturating relationships with the slopes of power functions (i.e., power coefficients) ranging between 0.15 and 0.32; the great majority of those studies used exactly the same variables used in this study [5]. Our analysis shows that among regions, despite environmental and historical differences, there was a similar non-saturating relationship between standing biomass and species richness, with markedly steeper slopes than previously reported in experimental studies. In fact, in seven of eight cases analyzed, the power coefficients were significantly greater than 1, indicating concave-up shapes for the relationship between standing biomass and richness. The hypothesis that the power coefficient was equal to 1 was rejected at *p*<0.05 in all cases, except for the relationship between biomass and species richness in the Indian Ocean (i.e., Pacific: power coefficient = 1.2, *R* ^2^ = 0.38; Indian: power coefficient = 1.1, *R* ^2^ = 0.58; Caribbean: power coefficient = 1.8, *R* ^2^ = 0.53; Eastern Pacific: power coefficient = 2.6, *R* ^2^ = 0.6; Figure S2). The slopes became steeper with the use of functional richness (Pacific: power coefficient = 2.3, *R* ^2^ = 0.38; Indian: power coefficient = 2.3, *R* ^2^ = 0.50; Caribbean: power coefficient = 3.0, *R* ^2^ = 0.44; Eastern Pacific: power coefficient = 4.3, *R* ^2^ = 0.4; Figures 2A–2D and S2; calculation of functional richness is detailed in Materials and Methods and Table S2; see Table S3 for comparison of different model fits). The steeper relationship with functional richness likely emerges from the fact that adding one functional group amounts to adding the biomass of multiple species. General trends were similar using species richness or functional richness (Figures S2 and S3); however, given the greater relevance of functional diversity to ecosystem functioning [21], for the remainder of the paper we describe the results using functional richness unless otherwise indicated.

In field studies, species or functional richness is likely to be correlated with abundance; hence, links between biomass and richness might not occur if differences in abundance are controlled. We assessed this possibility by standardizing our sampling sites to an equal number of individuals, so that the relationship between standing biomass and richness (whether of species or functional groups) could be assessed independently of variations in abundance. This reanalysis still yielded similar and strong relationships between standing biomass and richness (Figure S3). Using structural equation modeling to account for the effects of abundance and the environment on diversity, we found that functional richness retained a significant and independent effect on standing biomass in all regions (Figure 2M). Finally, we compared our patterns to a null model in which, from regional pools, we randomly selected individuals from randomly selected functional groups to equal the abundance of functional groups in each assemblage. This comparison demonstrated that the pattern between standing biomass and functional richness was unlikely to emerge by mere sampling of equally abundant functional groups from regional pools (red lines in Figure 2A–2D). The overall results of these analyses indicate that while abundance influences standing biomass, there is an equally strong and independent effect of diversity on standing biomass.

Non-saturating relationships between standing biomass and richness can arise through different mechanisms, some of which are supported by field or theoretical studies, but whose ultimate confirmation will require future studies. Theoretically, accelerating relationships between standing biomass and richness can occur when ecological interactions among species enhance their fitness in a given assemblage [22]–[25]. In the case of reef fishes, ecological interactions such as predation and competition can trigger faster somatic growth to gain a competitive advantage or to escape size-dependent predation [26],[27]. These ecological interactions can also cause early sexual maturation, leading to greater offspring production to compensate for higher mortality [26],[27]. Over evolutionary time, predation and competition can also lead to greater specialization, which favors faster somatic growth in feeding-specialized [28] and habitat-specialized [29] reef fishes. Coral reefs are structurally complex environments, which favor an even greater degree of specialization and more efficient use of narrower niches, making this type of ecosystem particularly likely to yield non-saturating relationships between richness and standing biomass. Non-saturating relationships may also reflect the effect of selective extinction of large species, which can be more extinction prone and more functionally efficient, therefore leading to more rapid declines in ecosystem functioning than random extinctions [30]. The effects of selective extinctions on ecosystem functioning can also be exacerbated because the lack of competitors of large species reduces the chance for compensatory responses or substitutability by other species [31]. Another possible mechanism is a “sampling effect” that results from the addition of species from a regional pool. However, our null model indicated that the link between standing biomass and richness is unlikely to result through simple sampling of equally abundant functional groups (red lines in Figure 2A–2D). Finally, the relationship between standing biomass and diversity could also be caused indirectly by factors not considered here. However, although we cannot refute this possibility, we made every effort to evaluate a broad range of variables known to affect reef fish assemblages. Regardless of the mechanism, the non-saturating relationship between standing biomass and diversity in reef fishes challenges the current paradigm emerging from experimental studies of a near-universal asymptotic relationship between ecosystem functioning and biodiversity [5]. It also confirms previous concerns about the extrapolation of experimental studies to predict the consequences of biodiversity loss in natural ecosystems [7]–[10] and implies that the true impact of biodiversity loss on ecosystem functioning may have been substantially underestimated [10].

**Figure 3:**
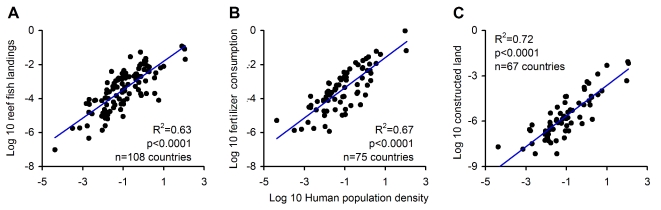
Surrogates for human population density near reefs. To assess the likely mechanism mediating the effect of human population density on reef fish biomass, we analyzed the relationships between human density near reefs and (A) fishing, (B) nutrient loads, and (C) habitat alteration. Fishing was estimated from reef fish landings reported to the Food and Agriculture Organization (http://www.fao.org/fishery/statistics/software/fishstat). For each country with coral reefs, we averaged reef fish landings between the years 1997 and 2001. Reef fish stocks were discriminated by classifying each of 1,472 stocks reported to the Food and Agriculture Organization as reef- or non-reef-associated using the Internet and other sources (http://www.fishbase.org). Nutrient load was quantified as fertilizer consumption using data obtained from the World Development Indicators database (http://www.worldbank.org/data). Finally, habitat alteration was quantified as the area of modified land indicated in the Global Land Cover 2000 dataset (http://ies.jrc.ec.europa.eu/global-land-cover-2000). Technical note: For purposes of comparison all variables were standardized by country area and area of reef. To control for type I errors arising from standardizing data by a common factor, significance levels were calculated by Monte-Carlo simulation, in which the slopes of the plots were calculated for each of 10,000 iterations in which standardization was done with random country areas and reefs, and then determining the fraction of “random” slopes above the true slope [51].

We evaluated the effect of human population density on standing biomass as a main effect and in interaction with functional diversity. We used human population density as a proxy for disturbances (see Figure 3), whereas its interaction with functional diversity was considered to test the possibility that biodiversity offers greater resilience or, conversely, more vulnerability to disturbances (see Materials and Methods). Among regions, the main effect of human population density was negative, although the fit, with the exception of the Eastern Pacific, was very poor (Figure 2E–2H). Accordingly, the main effect of human population density was not selected among the set of variables that “best” predict standing biomass (Figure 2M). In contrast, the interaction between functional richness and human density was selected in the “best” models for all regions (Figure 2M). This significant interaction can explain the overall weak effect of human population alone, as for the same number of people, standing biomass can be different, depending on local biodiversity. The overall pattern of the interaction between human density and biodiversity showed that standing biomass declined significantly from reefs with high diversity and low human density towards reefs with low diversity and large human populations (Figure 2I–2L). The implication of this interaction is that for the same number of people there is a larger reduction of biomass in more diverse systems; in other words, if one relates standing biomass to human density, the slope of this relationship becomes steeper among reefs of higher diversity (Figure S4). We presume that the stronger deleterious effect of human density on the functioning of diverse reefs can be related to the selective extinction of large species. Selective extinction of large species is well known in reef systems [17],[27],[32]. As mentioned before, the loss of large fish can lead to a rapid loss of ecosystem functioning [30], and their lack of competitors can prevent compensatory dynamics and/or substitutability by other species [31]. High-diversity systems may also be more vulnerable to human activities, as intrinsically more species and/or functional groups can be vulnerable to the large plethora of anthropogenic stressors. Independent of the mechanism, this result indicates that reef fish assemblages, particularly the most diverse, are greatly vulnerable to the effects of anthropogenic stressors (this may also be the case for corals [33]).

Finally, we found that marine protected area (MPA) effectiveness (see Materials and Methods for an explanation of this index) and environmental conditions were poor predictors of standing biomass, as neither one of these variables was selected for inclusion in the best models predicting standing biomass in all regions (Figure 2M). MPAs are a broadly recommended tool for halting declines in biodiversity [34]–[37], given their overwhelmingly positive effects on different ecological metrics, including standing biomass, as demonstrated in several meta-analyses (e.g., [36]). Our contrasting result about the effect of MPAs can be explained by different reasons. One possible explanation is statistical. Most studies on MPAs compare ecological conditions inside versus outside protected areas and/or before and after their establishment. A condition of these types of studies is that at this small scale the range of variation is always smaller than the range of variation that will be observed at a larger scale [38],[39]. As a consequence, the effect of MPAs can be reduced and at times lose its significance when compared to other factors in large-scale analyses [38]–[40]. Another possible explanation relates to the fact that MPAs are established mainly to address the effects of overfishing; however, other threats such as climate change, pollution, habitat loss, and invasive species can be just as deleterious [39]–[44] and are generally not (or cannot be) regulated within MPAs [37],[39],[40],[44]. This, in turn, would render moot any effect of MPAs, particularly over broad scales. The reduced effect of environmental variables on standing biomass was likely because the studied ecosystems occur in narrow regional tropical bands where environmental variation is low.

In this study, we used human population density as a generic proxy for anthropogenic disturbances given the availability of high-resolution and reliable data on human populations over the global domain examined. An important question, however, is, what particular human activity is responsible for the pattern described here? The effect of human density on standing biomass can operate through various mechanisms such as fishing, coastal development, and land use, each of which can result in deleterious effects on reef fish assemblages through overexploitation and the loss or degradation of habitats [32],[34]. To assess the human activities that may be responsible for the patterns described here, we compared human population density to proxies for fishing, coastal development, and land use (note: data on these measures were available only at the country level; Figure 3). Our results indicated that human density is highly and significantly related to the intensity of all three activities (Figure 3). Although the high collinearity among these proxies prevents us from making statistical inferences about causality, the fact that all proxies have been shown to affect reef fish assemblages [32],[34] suggests that the patterns described here may emerge through a combination of multiple human activities.

**Figure 4:**
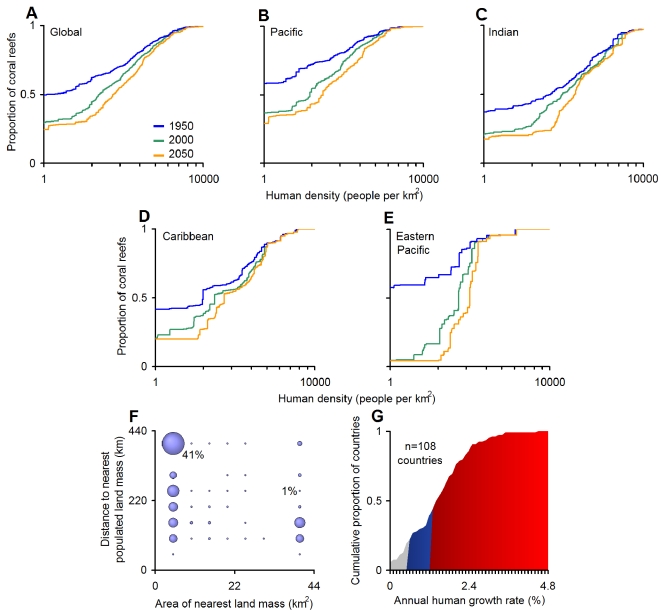
Human habitation of the world's coral reefs. Cumulative proportion of reefs located near human settlements (A) globally and (B–E) regionally. Data on coral reef areas were obtained from the Millennium Coral Reef Mapping Project (http://www.imars.usf.edu/MC/index.html; [37]). Plots in (A–F) are based on the division of the world's coral reefs into 5×5 km cells and the maximum human density occurring within a 50-km radius from the center of each cell. We used each country's growth rate between the years1950 and 2000 and that expected in 2050 under the United Nations Population Division World Population Prospects “medium variant” projection (see details at http://esa.un.org/unpp/) to calculate, for each reef cell, human density in 1950 and 2050, respectively. Plot in (F) depicts the proportion of the world's uninhabited coral reefs in the year 2000 (i.e., coral reef cells with zero humans within a 50-km radius) in terms of their distance to the closest human population center and the area of the nearest land. Plot in (G) describes current annual growth rates for countries with coral reefs as reported in the United Nations Population Division World Population Prospects (http://esa.un.org/unpp/). Growth rates that will cause doubling of human populations in >100, <100, and <50 years are shaded in grey, blue, and red, respectively.

The relevance of our results depends on the degree to which coral reefs, regionally and globally, are located near human settlements. Overlaying a map of the distribution of the world's human population on the global distribution of coral reefs, we found that in the year 2000, over 75% of the world's roughly 507,000 km^2^ of coral reefs were near (i.e., within 50 km of) human settlements (Figure 4A). This is a marked increase from the calculated 50% that were near human settlements in 1950 (Figure 4A). Given a moderate projection for human population growth, the proportion of coral reefs worldwide that will be close to human settlements may only increase to 76% by 2050 (Figure 4A). This small predicted increase over the next 40 years results from the fact that the current 25% of the world's uninhabited reefs are located at small and isolated locations (Figure 4F), where conditions for human habitation are harsh. The relative area of uninhabited reefs in 2000 varied from only 4% in the Eastern Pacific, to 17% in the Indian Ocean, 20% in the Caribbean, and 31% in the Pacific (Figure 4B–4E). The main effect of human population growth expected by 2050 is a greater density of people living near reefs (Figure 4A–4E). This effect may be exacerbated by urbanization, which is likely to accelerate in developing countries, particularly in coastal areas [45].

Coral reefs are one of the most diverse ecosystems on earth [46] and provide critical services to human welfare through the provision of food, tourism revenue, and coastal protection [17]. In this study, we have shown that the functioning of reef fish assemblages has a strong linkage with biodiversity and is being strongly and similarly shaped by human settlements worldwide. Our results also suggest that reef fish assemblages, particularly those most diverse, are highly vulnerable to the deleterious effects of human populations. Although presently uninhabited reefs will likely remain so in the near future, multiple stressors are associated with increasing human density (Figure 3), and countries with coral reefs are projected to double their human populations within the next 50 to 100 years, given their current rates of population growth (Figure 4G). This highlights the urgent need to implement comprehensive reef governance at local, regional, and global scales to maintain biodiversity and confront the variety of drivers and stressors associated with coastal habitation, as well as long-term strategies (improvements in education, empowerment of women, family planning, poverty alleviation, etc.) to curb the growth of coastal human populations. Policy tools that address the socioeconomic roots of overfishing, biodiversity loss, and reef degradation [47] are clearly necessary.

## Materials and Methods

### Biological Databases

Data on reef fish assemblages were obtained for 6,142 sampling units, of which 98% were transects and the remaining point counts, in 1,906 reef locations worldwide (Table S1). We considered only surveys that sampled all or almost all species, their abundances, and their body sizes. With the exception of two locations in the Pacific Ocean, where only species in the 15 most common families were sampled, all data included all detectable species (we excluded gobies and small blennies because of taxonomic difficulties in identifying and sighting these species).

We analyzed data from the fore reef or, when habitat information was not available, within depths of 7 to 17 m. All sampled units were standardized to 50 m^2^ by randomly sub-sampling the individuals occurring within the fraction of the sampled unit that equaled 50 m^2^. For sites sampled with units smaller than 50 m^2^, units were aggregated until the total area sampled equaled 50 m^2^. Data on diversity and biomass (see below) were calculated for each sampling unit and averaged at the location level. Because all data were collected by experienced researchers, sampling errors in our census data due to differences among observers are expected to be negligible.

### Assemblage Diversity and Standing Biomass

The global database comprised 2,036 species, all of which were verified taxonomically using Fishbase (http://www.fishbase.org). Biodiversity was calculated as species richness and functional richness (i.e., the number of functional groups resulting from assigning individual fishes to one of seven feeding groups: large predator, piscivore-invertebrate feeder, planktivore, colonial invertebrate feeder, benthic herbivore, omnivore, or detritivore). Data on food items were primarily obtained from Fishbase and other Internet sources and were used in a classification scheme (see Table S2) to assign each species to a feeding group. For species lacking data on food items, we assigned the most common feeding group of the species in the same genus, or family when no genus data were available. Feeding attributes have been used in contemporary research to assess functional richness in reef fishes, given their relation to energy flow and rates of biomass turnover in the food web [32]. Although we lack data on other traits for most reef fish species, using more elaborate classification schemes and post hoc selection of traits that best predict ecosystems' responses would be expected to yield stronger relationships than the ones reported here [48].

Body mass for individual fishes (*W*) was calculated using the allometric length–weight conversion as *W = aL^b^*, where *L* is the body length of each individual, and parameters *a* and *b* are constants for each species (data from http://www.fishbase.org). In turn, standing biomass was quantified as the cumulative weight of fishes in each sampling unit.

### Anthropogenic and Environmental Databases

For each site, we gathered data on the mean and standard deviation of sea surface temperature (data were collected at 4-km resolution by the AVHRR Pathfinder Version 5 SST Project, which provides annual mean and standard deviation values for the years 1985 to 2001; http://www.nodc.noaa.gov/SatelliteData/pathfinder4km/userguide.html), ocean primary productivity (derived from chlorophyll-a concentrations estimated from remote sensors at a 9-km resolution and averaged between the years 1997 and 2001; http://oceancolor.gsfc.nasa.gov/cgi/l3), reef isolation (calculated as the shortest distance from each site to a continent or any island larger than 10,000 km^2^), reef area (calculated as the area of reefs within a 5-km radius of each site; data source described below), the yearly frequency with which hurricanes of categories 1 to 5 passed within a 50-km radius of each site (data between the years 1990 and 2000 from http://weather.unisys.com/hurricane), the maximum human density occurring within a 25-km radius of each site (data at 0.25° cells for the year 2000: http://sedac.ciesin.columbia.edu/gpw/global.jsp), and an index of MPA effectiveness that integrated protection properties such as the presence of an MPA, the extent of no-take regulations, levels of poaching, size and number of years since the establishment of the MPA, and level of risk due to external threats (source: Mora et al. [37]).

### Statistical Methods

We used the software Analysis of Moment Structures (AMOS) [49] to fit structural equation models to the relationship between standing biomass and functional richness while considering the covariance between functional richness and abundance and the effects of other variables. We considered all environmental variables as one latent variable that included the simultaneous effect of the mean and standard deviation of sea surface temperature, ocean primary productivity, reef isolation, reef area, and the frequency of hurricanes. We also analyzed the independent effect of MPA effectiveness. Additionally, we tested the effect of human density as a main effect and in interaction with biodiversity. We assessed the interaction between human density and biodiversity to test the prediction that biodiversity offers greater resilience [32] or, conversely, more vulnerability [30],[31] to the effects of human stressors. Statistically, this interaction was evaluated by including the interaction term between diversity and human population density (i.e., human density multiplied by diversity) in the structural equation model. Finally, we considered the potential correlation between abundance and functional richness. The outline of the structural equation model is presented in Figure 2M. To avoid over-fitting of the models, we implemented a process of selection of variables that improved the overall goodness-of-fit of the structural equation model for each region. We used as metrics of goodness-of-fit the goodness-of-fit index and the Chi-square divided by the degrees of freedom. The former metric is analogous to the coefficient of determination in regression analysis, with a value of 1 representing perfect fit [49],[50]. The latter metric quantifies the tradeoff in the model between fit and parsimony with a “reasonable good model” varying between values of 5 and 1 [49]. For each region, we started with the full model (arrows in Figure 2M) and conducted sequential removal of variables that improved fit until the criteria of “good” fit defined above was achieved.

## Supporting Information

Figure S1 **Standing biomass as a surrogate of ecosystem processes.**
(1.25 MB DOC)

Figure S2 **Patterns of standing biomass and species and functional richness in coral reef fishes.**
(0.13 MB DOC)

Figure S3 **Effect of the number of individuals on the relationship between diversity and standing biomass.**
(0.14 MB DOC)

Figure S4 **Interpretation of the significant interaction between human density and biodiversity.**
(0.07 MB DOC)

Figure S5 **Changes in standing biomass along gradients of human density and biodiversity.**
(0.27 MB DOC)

Table S1 **Description of the assembled database.**
(0.05 MB DOC)

Table S2 **Scheme for classification of species to functional feeding groups.**
(0.05 MB DOC)

Table S3 **Fit of linear, polynomial, exponential, and power models to the relationship between standing biomass as the dependent variable and functional richness as the independent variable.**
(0.06 MB DOC)

Table S4 **Data used to establish the link between energy consumption and body mass in fishes.**
(0.25 MB DOC)

Table S5 **Data used to establish the link between biomass production and body mass in fishes.**
(0.10 MB DOC)

Text S1 **Sources of data.**
(0.07 MB DOC)

## Abbreviations

MPA: marine protected area
