# Supplementary material for: Global Human Footprint on the Linkage between Biodiversity and Ecosystem Functioning in Reef Fishes
Source: PLoS Biol. 2011 Apr 5;9(4):e1000606. doi: 10.1371/journal.pbio.1000606 (PMC3071368; doi:10.1371/journal.pbio.1000606)
Supplement: Figure S1 — Standing biomass as a surrogate of ecosystem processes. (1.25 MB DOC) [file pbio.1000606.s001.doc]

**Figure S1. Standing biomass as a surrogate of ecosystem processes.**

*Theoretical demonstration*

The relationship between an individual body mass [***Mi***] and several of its energetic processes [***Pi***] such as mass production, energy consumption, development rate, and others [see detailed review in Brown et al 2004 and table embedded in figure below] is well defined with a power model of the form:


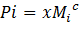
 [1] [See plot **a**]

where ***x*** is a normalization constant and ***c*** is the allometric exponent [usually ***c*** ≈ ¾] [see e.g., Brown et al. 2004 and table embedded in figure below]. The allometric scaling of body mass to energetic processes arises from the intrinsic dependence between the energetic requirements to fuel metabolism and body size [e.g. Peters 1986, Brown et al. 2004].

In turn, the biomass production of an entire assemblage [***BP***] can be quantified as the cumulative biomass production of the constituent individuals as:


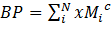
 or
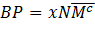
 [2]

Where ***N*** is the number of individuals and
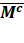
 is the average biomass production of the individuals in the assemblage. Similarly, standing biomass [***SB***] can be measure as the cumulative body mass of the individuals in the assemblage. That is:


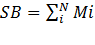
 or
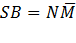
 [3]

Where
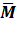
 is the mean body mass of the individuals in the assemblage.

There is no simple relation between standing biomass, ***SB***, and biomass production of an assemblage, ***BP***, because generally
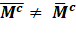
. However, the average production of the individuals in the assemblage,
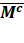
, can be approximated as a function of mean body mass,
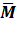
, by Taylor expanding
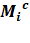
 around
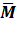
, ignoring terms of order 3 and higher, and then taking the mean of the second-order approximation. This yields:


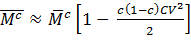
 [4]

Where ***CV*** is the coefficient of variation of the body masses of the individuals in the assemblage. By merging equations 2 and 4, biomass production at the assemblage level, ***BP***, can be approximate as:


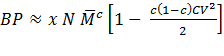
 [5]. Since
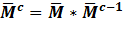
 , then equation 5 can be rewritten as:


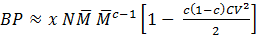
 [6]; and since
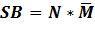
 [i.e. Eq.3], then equation 6 can be simplified as:


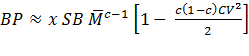
 [7].

Equation 7 reflects the biomass production of a system, ***BP***, as a function of its standing biomass***, SB***, and the allometric escalation of individual body mass to several energetic processes [i.e. Eq. 1] [see plot **c** and simulations below].

*Simulations*

We used different scenarios to indicate the range of conditions over which standing biomass reliably surrogates assemblage-level processes, which are related to body size at the individual-level. We started by creating different abundance/size distributions [plot **a**]. From these underlying distributions, we randomly sampled without replacement individuals to create 49 assemblages varying in abundance from 2 to 1500 individuals. For each assemblage, we calculated the cumulative mass of their constituent individuals [i.e. standing biomass using equation 3] and the cumulative sum of the process [e.g. biomass production using equation 2] calculated for each individual through the power law defined in equation 1. We simulated an extreme range of variation in the allometric exponent from 0 to 1. Plot **b** shows two examples of simulated processes related to body size by a power law with allometric exponents of 0.8 and 0.6. Plot **c** shows the relationship between standing biomass and the cumulative assemblage-process assuming the abundance/size distributions outlined in plot **A** and the individual-level power laws defined in plot **b**. As demonstrated in equation 7, we found that the relationship between assemblage level-processes such a biomass production and standing biomass are remarkably similar among different underlying abundance/size distributions and are precisely predicted with the parameters of the allometric relationships [plot **c** to **e**]. R^2^-values for relationships were above 99% in all underlying abundance/size distributions and allometric coefficients [plot **e**]. For reference, we provide individual level processes related to body mass in fishes [plot **f-g**] [Data collected from different sources and presented in Table S4 and S5] and their subsequent relationship at the assemblage level using our data for reef fishes in the Caribbean [plot **h**, **i**]. We should note that at least one other independent study [i.e. Savage et al. 2004] has found that individual- and assemblage-level processes are “*inextricably linked because metabolism sets both the demand for environmental resources and the resource allocation to survival, growth, and reproduction*”. These results altogether suggest that standing biomass is a good surrogate of community processes over a broad range of scenarios. Note that the analyses and data shown here do not include temperature, which is known to affect metabolism [Brown et al 2004] and which may account for some of the unexplained variation in plots **f** and **g**. The predictable effect of temperature on metabolism [Brown et al. 2004] could also be incorporated into this theory; however, in the case of coral reef fishes the effect of temperature on ecosystem processes is likely to be small or homogeneous given the tropical limited distribution of this ecosystem.

**
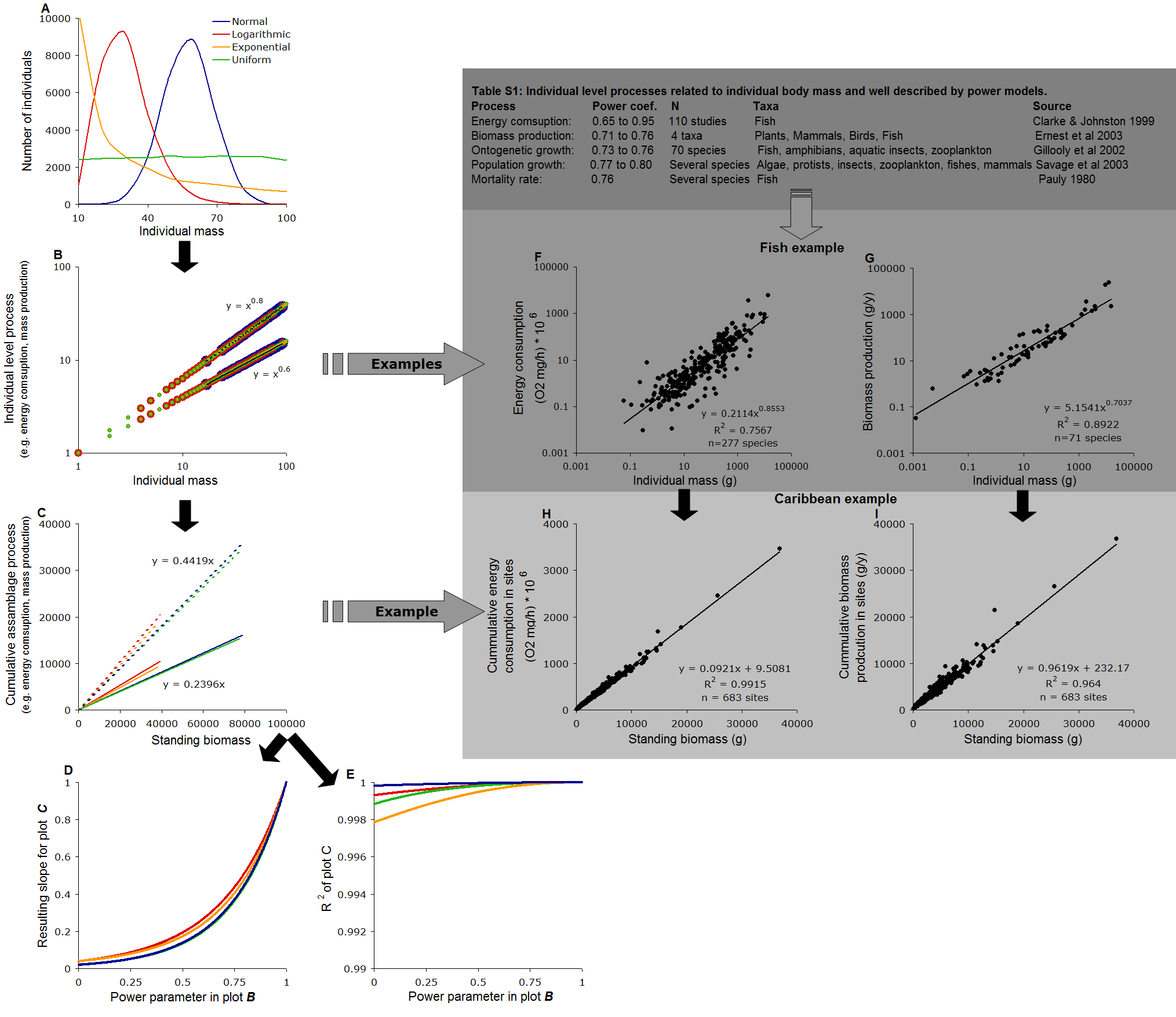
**
