# Supplementary material for: Global Human Footprint on the Linkage between Biodiversity and Ecosystem Functioning in Reef Fishes
Source: PLoS Biol. 2011 Apr 5;9(4):e1000606. doi: 10.1371/journal.pbio.1000606 (PMC3071368; doi:10.1371/journal.pbio.1000606)

**Figure S2. Patterns of standing biomass and species and functional richness in coral reef fishes.** All relationships were significant at *P*<0.01.


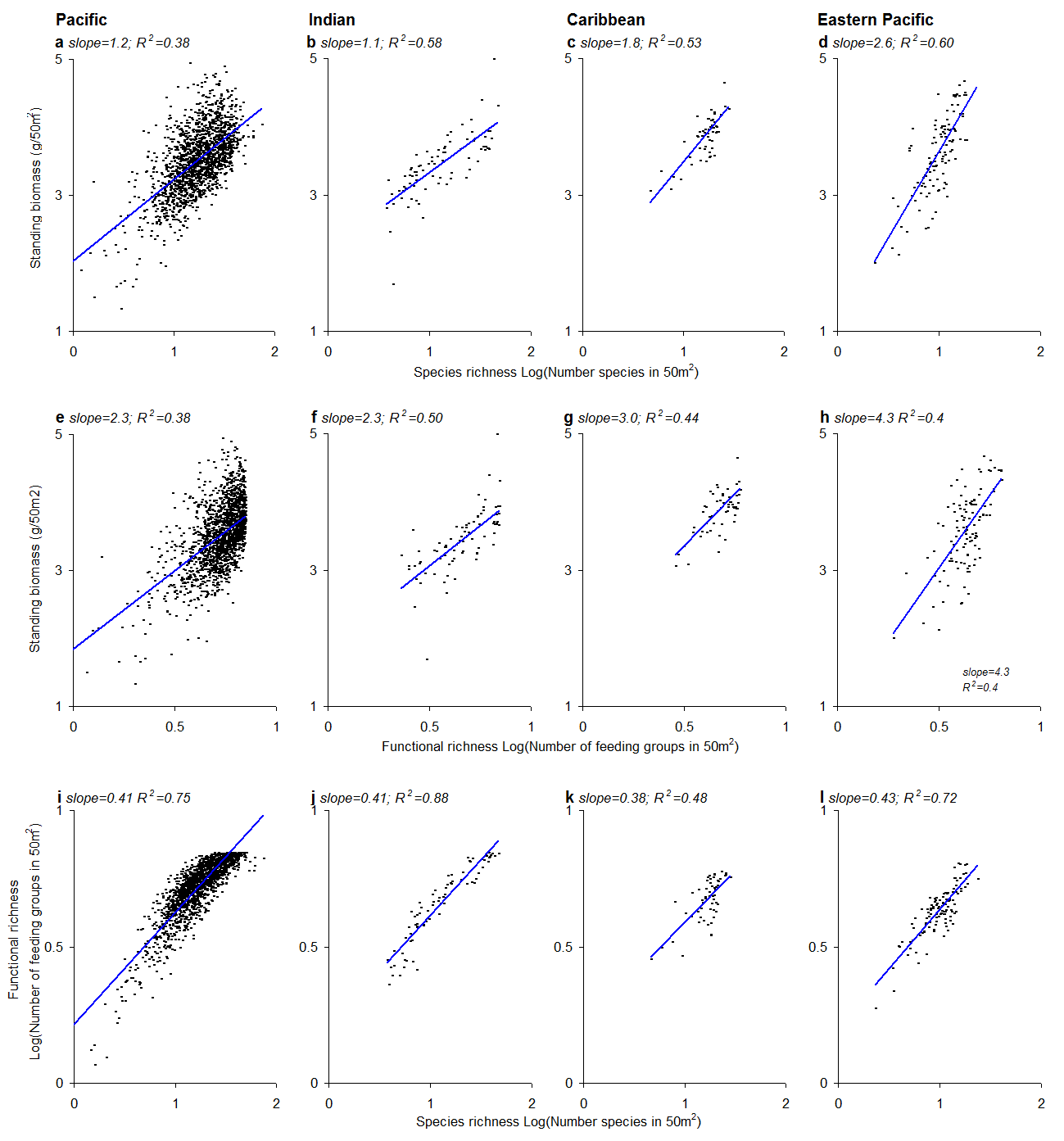

Supplement: Figure S2 — Patterns of standing biomass and species and functional richness in coral reef fishes. (0.13 MB DOC) [file pbio.1000606.s002.doc]
