# Supplementary material for: Global Human Footprint on the Linkage between Biodiversity and Ecosystem Functioning in Reef Fishes
Source: PLoS Biol. 2011 Apr 5;9(4):e1000606. doi: 10.1371/journal.pbio.1000606 (PMC3071368; doi:10.1371/journal.pbio.1000606)
Supplement: Figure S3 — Effect of the number of individuals on the relationship between diversity and standing biomass. (0.14 MB DOC) [file pbio.1000606.s003.doc]

**Figure S3. Effect of the number of individuals on the relationship between diversity and standing biomass.** Different attributes of communities are inherently correlated with each other. In our case, for instance, the presence of a certain number of individuals in a community will account for some standing biomass and these same individuals are likely to belong to a certain number of species and functional groups. As such, one could potentially find a relationship between standing biomass and richness that is indirectly caused by the number of individuals. To assess this possibility, we reanalyzed our patterns by standardizing locations by a constant number of individuals. For each location, we randomly selected 100 individuals [we chose this number because resampling a larger number of animals reduced considerably the number of locations for which the analysis could be done] and calculated the number of species, feeding groups and their total biomass. This approach was repeated 1000 times to get an average species richness, functional richness and standing biomass of 100 randomly selected fish at each location. We used these variables to reassess our patterns between diversity and standing biomass. In this case, the effect on the response variable [i.e. biomass] is truly due to diversity as the number of individuals was held constant. The results indicate that the relationships between richness and biomass presented in our paper are robust to the effects of variations in the number of individuals
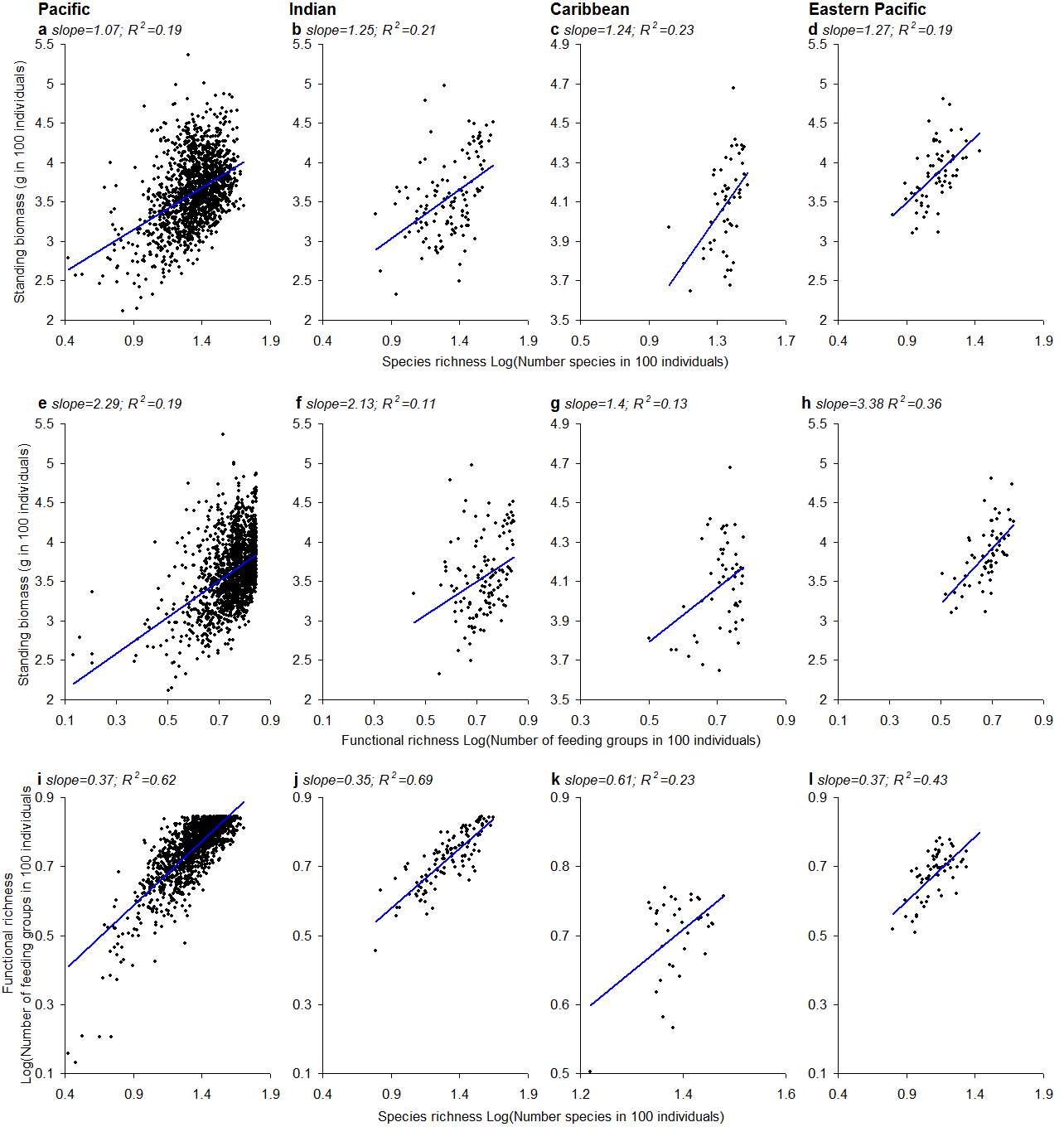
among locations. All relationships were significant at *P*<0.05.
