# Supplementary material for: Global Human Footprint on the Linkage between Biodiversity and Ecosystem Functioning in Reef Fishes
Source: PLoS Biol. 2011 Apr 5;9(4):e1000606. doi: 10.1371/journal.pbio.1000606 (PMC3071368; doi:10.1371/journal.pbio.1000606)
Supplement: Figure S4 — Interpretation of the significant interaction between human density and biodiversity. (0.07 MB DOC) [file pbio.1000606.s004.doc]

**Figure S4. Interpretation of the significant interaction between human density and biodiversity.** For each region, the effect of functional richness (*R*) and human density (*H*) on standing biomass (*B*) can be modeled using the significant covariance estimates obtained from the Structural Equation Model (SEM) presented in Figure 2M as:

*B_RH_ = a_1_R – a_2_RH*

*Note: the full prediction of standing biomass requires an intercept plus the additional covariance estimates presented in the SEM of each region, which are omitted here as the interest is to interpret only the interaction between biodiversity and human density.*

For the Pacific, as an example, the effect of functional richness and the interaction between human density and richness on standing biomass is:

*B_RH_ = 5.091R – 0.261RH*

To illustrate the interaction between human density and functional richness, we plotted the relationship between standing biomass and human density for the lowest and highest functional diversity we observed in the Pacific. That is:


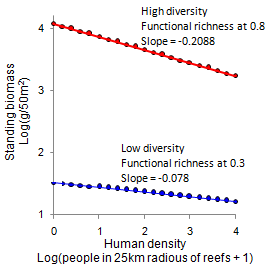


Note that the rate of decline in standing biomass (i.e., the slope) is markedly steeper at high compared to low diversity, which illustrates the nature of the significant negative interaction between human density and richness on standing biomass.

Below we show the slopes of the relationships between standing biomass and human density across the gradient of functional richness in each region.


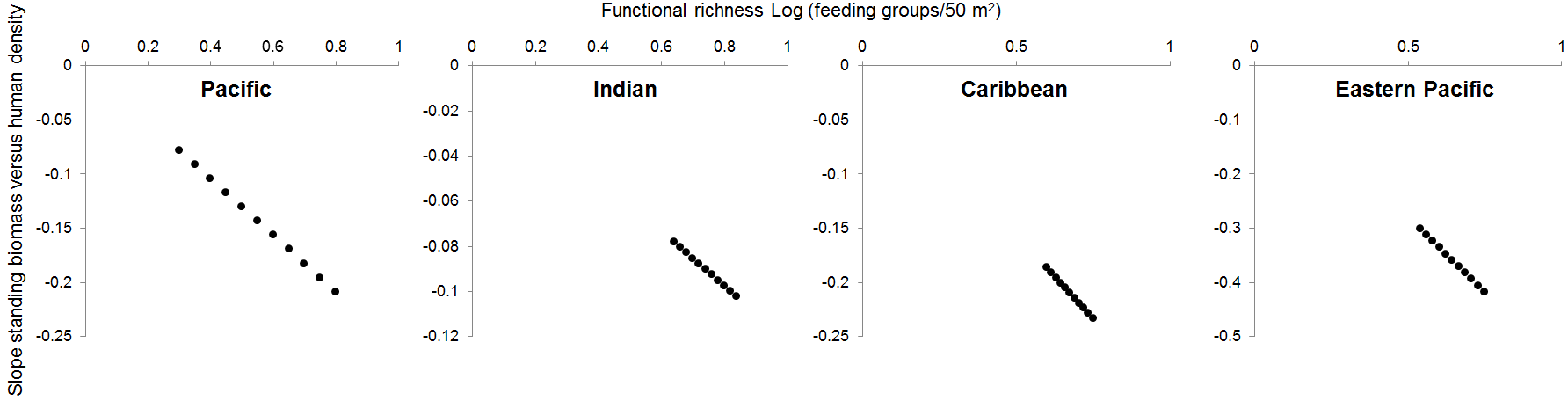


These plots show that the slopes of the relationship between standing biomass and human density become steeper in more diverse systems; in other words, the rate of biomass decline due to human density is significantly larger in more diverse reefs.
