# Supplementary material for: Global Human Footprint on the Linkage between Biodiversity and Ecosystem Functioning in Reef Fishes
Source: PLoS Biol. 2011 Apr 5;9(4):e1000606. doi: 10.1371/journal.pbio.1000606 (PMC3071368; doi:10.1371/journal.pbio.1000606)
Supplement: Figure S5 — Changes in standing biomass along gradients of human density and biodiversity. (0.27 MB DOC) [file pbio.1000606.s005.doc]

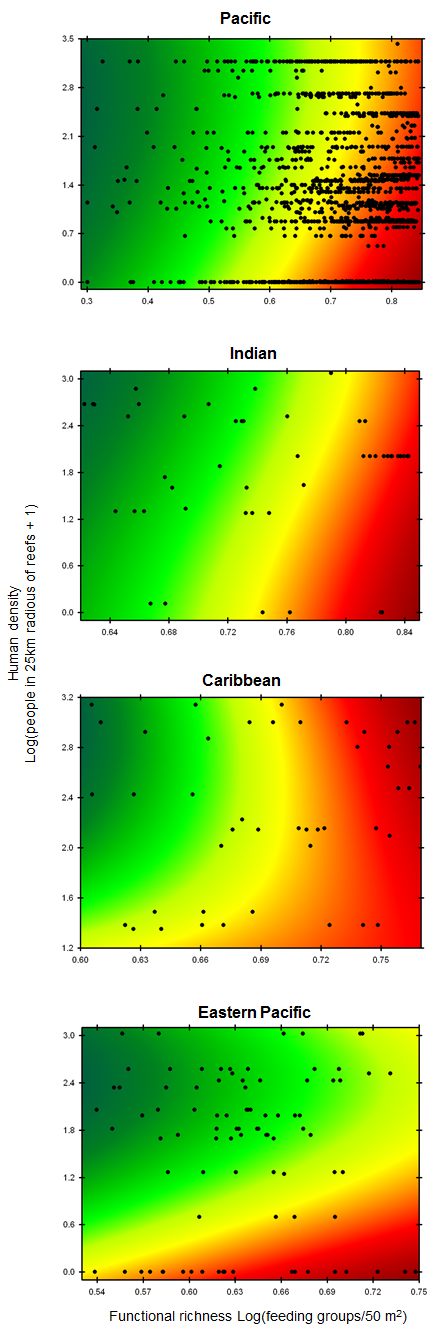
**Figure S5. Changes in standing biomass along gradients of human density and biodiversity.** These plots are the same as in Fig. 2**e-h** with the difference that here the surface trends were fitted to the raw data. As mentioned in the text, for field studies it is critical to examine specific relationships after accounting for potential confounding variables. As such, the predicted effects of biodiversity and human population density on standing biomass were quantified and presented in Fig. 2**e-h** after controlling for the confounding effects of abundance, environmental variables and MPAs. Here, for information purposes, we present the effects of biodiversity and human population density on standing biomass as depicted from the raw data and potentially confounded by other variables.
