# Supplementary material for: Global Human Footprint on the Linkage between Biodiversity and Ecosystem Functioning in Reef Fishes
Source: PLoS Biol. 2011 Apr 5;9(4):e1000606. doi: 10.1371/journal.pbio.1000606 (PMC3071368; doi:10.1371/journal.pbio.1000606)
Supplement: Table S1 — Description of the assembled database. (0.05 MB DOC) [file pbio.1000606.s006.doc]

Table S1. Description of the assembled database.

| **Region** | **Countries or states** | **Contacts** | **Number of locations** | **Number of sampling units** |
| --- | --- | --- | --- | --- |
| Caribbean | Belize, Cuba, Jamaica, Mexico, United States, Venezuela. | Gustavo Paredes, Juan Cruz | 56 | 198 |
| Indian | Australia, Indonesia, Israel, Madagascar, Maldives, Oman, Reunion, Seychelles, Tanzania, United Arab Emirates. | Graham Edgar, Maria Beger, Eran Brokovich, Charlie Gough, Laurent Vigliola, Paolo Usseglio, Pascale Chabanet, Nick Graham. | 78 | 717 |
| Pacific | American Samoa, Australia, Fiji, French Polynesia, Guam, Hawaii, Howland Island, Indonesia, Japan, Jarvis Island, Johnston Atoll, Kiribati, Marshall Islands, Micronesia, New Caledonia, Northern Mariana Islands, Palau, Palmyra Atoll, Papua New Guinea, Philippines, Solomon Islands, Tonga, United States, Wake Island, Wallis and Futuna. | Alison Green, Ivor Williams, David Booth, Graham Edgar, Michel Kulbicki, Shaun Wilson, Andrew Brook, Sebastian Ferse, Yohei Nakamura, Stuart Sandin, Maria Beger, Mark Tupper, Laurent Wantiez, Nick Graham, Tau Morove. | 1658 | 4800 |
| Eastern Pacific | Colombia, Costa Rica, Ecuador, Mexico, Nicaragua, Panama. | Edgar Graham, Arturo Ayala, Octavio Aburto. | 114 | 427 |
