# Supplementary material for: Global Human Footprint on the Linkage between Biodiversity and Ecosystem Functioning in Reef Fishes
Source: PLoS Biol. 2011 Apr 5;9(4):e1000606. doi: 10.1371/journal.pbio.1000606 (PMC3071368; doi:10.1371/journal.pbio.1000606)
Supplement: Table S2 — Scheme for classification of species to functional feeding groups. (0.05 MB DOC) [file pbio.1000606.s007.doc]

Table S2. Scheme for classification of species to functional feeding groups

| **Feeding group** | **Description** | **FishBase food item** | | |
| --- | --- | --- | --- | --- |
|  |  | They always eat | They eat at times | They never eat |
| Large predator | Fish that eats other fish and/or mobile invertebrates. As adults they attain >50 cm body length. | Nekton ***and/or***  Zoobenthos | Zooplankton | Benthic algae/weeds ***and/or***  Phytoplankton ***and/or***  Detritus ***and/or***  Colonial invertebrates |
| Piscivore-invertebrate feeder | Fish that eats other fish and/or mobile invertebrates. As adults they attain <50 cm body length. | Nekton ***and/or***  Zoobenthos | Zooplankton | Benthic algae/weeds ***and/or***  Phytoplankton ***and/or***  Detritus ***and/or***  Colonial invertebrates |
| Planktivore | Fish that eats zooplankton and phytoplankton | Phytoplankton ***and/or***  Zooplankton |  | Benthic algae/weeds ***and/or***  Detritus ***and/or***  Colonial invertebrates |
| Colonial invertebrate feeder | Fish that eats corals and sponges | Colonial invertebrates | Detritus | Nekton ***and/or***  Zoobenthos ***and/or***  Benthic algae/weeds |
| Benthic herbivore | Fish that eats only algae. These differ from an herbivore feeding upon phytoplankton, which was classified as “Planktivore” | Benthic algae/weeds | Benthic algae/weeds ***and/or***  Zooplankton ***and/or***  Detritus | Nekton ***and/or***  Zoobenthos ***and/or***  Phytoplankton |
| Omnivore | Fish that eats a variety of food items, including a mixture of animals, plants, and/or detritus | Animals  Nekton ***and/or***  Zoobenthos ***and/or***  Zooplankton ***and/or***  Colonial invertebrates ***and/or***  Plants  Benthic algae/weeds ***and/or***  Phytoplankton  Detritus  Detritus | Colonial invertebrates ***and/or***  Detritus | Nekton ***and/or***  Zoobenthos |
| Detritivore | Fish that eats small living and non-living particles | Detritus |  |  |

Nekton: This includes food items defined in Fishbase as “Finfish” and “Cephalopods”

Zoobenthos: This includes food items defined in Fishbase as “Benth. crust.”, ***“***Mollusks”, ***“***Echinoderms”, “Worms”, and ***“***Other benth. Invertebrates”.

Zooplankton: This includes food items defined in Fishbase as “Plankt. Crust” and “Other plank. Invertebrates”.

Detritus: This includes food items defined in Fishbase as “Detritus” and “Periphyton”, which refers to mixtures of microscopic algae, cyanobacteria, and/or heterotrophic microbes.

Colonial invertebrates: This includes food items defined in Fishbase as “Cnidarians” and “Sponges/tunicates”.
