# Supplementary material for: Global Human Footprint on the Linkage between Biodiversity and Ecosystem Functioning in Reef Fishes
Source: PLoS Biol. 2011 Apr 5;9(4):e1000606. doi: 10.1371/journal.pbio.1000606 (PMC3071368; doi:10.1371/journal.pbio.1000606)
Supplement: Table S3 — Fit of linear, polynomial, exponential, and power models to the relationship between standing biomass as the dependent variable and functional richness as the independent variable. (0.06 MB DOC) [file pbio.1000606.s008.doc]

Table S3. Fit of linear, polynomial, exponential and power models to the relationship between standing biomass as the dependent variable and functional richness as the independent variable. The parameters of the different models in each region and their coefficients of determination are shown in the table below. The power function yielded the best fits to the patterns in all regions [compare coefficients of determination]. Note that the power coefficients [bold numbers] in all regions were larger than 1 indicating a concave-up relationship.

|  | Pacific | Indian | Caribbean | Eastern Pacific |
| --- | --- | --- | --- | --- |
| Linear model  Biomass = A * functional richness + B | | | | |
| A | -4235 | -8726 | -35874 | -26758 |
| B | 1850 | 2906 | 1017 | 8168 |
| R^2^ | 0.05 | 0.13 | 0.09 | 0.27 |
| Polynomial second order  Biomass=A*functional richness+ B*functional richness^2^ + C | | | | |
| A | 18.55 | -6544 | -59757 | -15686 |
| B | 188 | 989 | 7671 | 2694 |
| C | -55 | 11908 | 118140 | 23921 |
| R^2^ | 0.06 | 0.16 | 0.13 | 0.31 |
| Polynomial third order  Biomass=A*functional richness+ B*functional richness^2^ + C* functional richness^3^ + D | | | | |
| A | 718 | 4530 | 59568 | -15659 |
| B | 15.14 | -1503 | -143342 | 2687 |
| C | 13.1 | 176.86 | 11304 | 0.53 |
| D | -885 | -3479 | -803244 | 23886 |
| R^2^ | 0.06 | 0.16 | 0.18 | 0.31 |
| Exponential Model  Biomass = A * exp [functional richness * B] | | | | |
| A | 153.5 | 0.53 | 292.36 | 39.7 |
| B | 0.56 | 210.2 | .68 | 1.05 |
| R^2^ | 0.32 | 0.50 | 0.43 | 0.44 |
| Power model  Biomass = A * functional richness ^ B | | | | |
| A | 70.46 | 77.03 | 68.24 | 7.7 |
| B | **2.3** | **2.3** | **3.04** | **4.3** |
| R^2^ | 0.38 | 0.52 | 0.44 | 0.44 |
