# Supplementary material for: Global Human Footprint on the Linkage between Biodiversity and Ecosystem Functioning in Reef Fishes
Source: PLoS Biol. 2011 Apr 5;9(4):e1000606. doi: 10.1371/journal.pbio.1000606 (PMC3071368; doi:10.1371/journal.pbio.1000606)
Supplement: Table S4 — Data used to establish the link between energy consumption and body mass in fishes. (0.25 MB DOC) [file pbio.1000606.s009.doc]

Table S4. Data used to establish the link between energy consumption and body mass in fishes [see Fig. S1f]. Data were obtained from www.Fishbase.org on April 23, 2010.

| **Species Name** | **Weight [g]** | **Energy consumption [O2 mg/h]** |
| --- | --- | --- |
| *Abramis brama* | 312.96 | 136.7635 |
| *Acanthopagrus schlegelii schlegelii* | 254 | 5.0292 |
| *Acipenser gueldenstaedtii* | 208 | 17.264 |
| *Acipenser nudiventris* | 89 | 13.1987 |
| *Acipenser ruthenus* | 304 | 105.5488 |
| *Acipenser stellatus* | 10400 | 882.96 |
| *Acipenser transmontanus* | 950 | 4.085 |
| *Aequidens pulcher* | 5 | 0.4035 |
| *Alburnus alburnus* | 13 | 3.2591 |
| *Ambassis interrupta* | 5 | 0.268 |
| *Ameiurus melas* | 50 | 4.76 |
| *Ameiurus natalis* | 23 | 1.3915 |
| *Ameiurus nebulosus* | 333 | 115.218 |
| *Anabas testudineus* | 60 | 0.276 |
| *Anguilla anguilla* | 1191 | 97.7811 |
| *Anguilla australis australis* | 700 | 15.19 |
| *Anguilla japonica* | 325 | 1.95 |
| *Anguilla rostrata* | 315 | 10.4265 |
| *Anoplogaster cornuta* | 50.9 | 9.3147 |
| *Aphanius dispar dispar* | 0.79 | 0.319397 |
| *Arapaima gigas* | 3040 | 27.056 |
| *Aristostomias lunifer* | 21.1 | 1.60993 |
| *Astatotilapia burtoni* | 15.2 | 0.6764 |
| *Astronotus ocellatus* | 345 | 3.795 |
| *Bajacalifornia burragei* | 24.9 | 0.94869 |
| *Balistes capriscus* | 700 | 90.09 |
| *Bathylagus stilbius* | 8.55 | 1.56465 |
| *Bathylagus wesethi* | 1.5 | 0.55005 |
| *Borostomias panamensis* | 110.25 | 14.2884 |
| *Brevoortia tyrannus* | 80.9 | 16.26899 |
| *Callionymus lyra* | 150 | 34.065 |
| *Campostoma anomalum* | 19.25 | 4.329325 |
| *Caranx hippos* | 38.3 | 14.01014 |
| *Carassius auratus auratus* | 182.5 | 22.13725 |
| *Carassius carassius* | 60 | 9.75 |
| *Catostomus commersonii* | 282 | 59.22 |
| *Catostomus tahoensis* | 53.09 | 9.641144 |
| *Centropristis striata* | 108 | 12.8628 |
| *Chaenocephalus aceratus* | 1130 | 158.087 |
| *Channa marulia* | 93 | 1.6368 |
| *Channa orientalis* | 65 | 0.598 |
| *Channa punctata* | 102.33 | 4.50252 |
| *Channa striata* | 82 | 1.5416 |
| *Channichthys rhinoceratus* | 200 | 67.46 |
| *Chanos chanos* | 11 | 0.3377 |
| *Chiloscyllium plagiosum* | 880 | 31.944 |
| *Chromis chromis* | 13.2 | 3.83988 |
| *Cichlasoma bimaculatum* | 83 | 3.1042 |
| *Cirrhinus cirrhosus* | 1821 | 72.6579 |
| *Citharichthys stigmaeus* | 15 | 1.8945 |
| *Clarias batrachus* | 95.7 | 4.6893 |
| *Clinocottus analis* | 37 | 4.514 |
| *Colisa fasciata* | 12 | 1.9488 |
| *Colossoma macropomum* | 2100 | 62.79 |
| *Conger conger* | 545 | 90.034 |
| *Coregonus autumnalis* | 234 | 124.371 |
| *Coregonus fera* | 0.11 | 0.11506 |
| *Coregonus sardinella* | 365 | 118.1505 |
| *Coryphaena equiselis* | 0.43 | 7.625663 |
| *Coryphaena hippurus* | 0.9 | 0.74295 |
| *Cottus gobio* | 2.9 | 1.22728 |
| *Ctenopharyngodon idella* | 16.05 | 1.491045 |
| *Cubiceps whiteleggii* | 1.3 | 1.55012 |
| *Cyprinella lutrensis* | 3 | 2.4972 |
| *Cyprinodon variegatus variegatus* | 2.4 | 0.27 |
| *Cyprinus carpio carpio* | 3487 | 665.6683 |
| *Dactylopterus volitans* | 0.06 | 0.176196 |
| *Dasyatis sabina* | 859 | 75.8497 |
| *Diaphus theta* | 2.65 | 2.16293 |
| *Dimidiochromis compressiceps* | 11.1 | 1.54623 |
| *Diplodus sargus sargus* | 25.75 | 8.484625 |
| *Dorosoma cepedianum* | 256.9 | 70.9044 |
| *Echeneis naucrates* | 487 | 39.447 |
| *Echiichthys vipera* | 10.5 | 2.7531 |
| *Embiotoca lateralis* | 599 | 66.1895 |
| *Encheliophis homei* | 7.5 | 0.28575 |
| *Engraulis japonicus* | 634.35 | 356.9487 |
| *Epinephelus akaara* | 281 | 8.3738 |
| *Eretmodus cyanostictus* | 6.4 | 0.9568 |
| *Erimyzon oblongus* | 25.8 | 4.49952 |
| *Erpetoichthys calabaricus* | 27.4 | 1.7673 |
| *Esomus danricus* | 0.62 | 0.162626 |
| *Esox lucius* | 1850 | 189.81 |
| *Esox masquinongy* | 19.2 | 11.98464 |
| *Etheostoma blennioides* | 12.35 | 3.234465 |
| *Etheostoma boschungi* | 1.2 | 0.11328 |
| *Etheostoma duryi* | 2 | 0.184 |
| *Etheostoma flabellare* | 1.4 | 0.10948 |
| *Etheostoma fusiforme* | 0.6 | 0.07602 |
| *Etheostoma rufilineatum* | 4.3 | 0.3354 |
| *Etheostoma squamiceps* | 1.7 | 0.16507 |
| *Euthynnus affinis* | 2260 | 815.182 |
| *Exodon paradoxus* | 4.77 | 0.270936 |
| *Fundulus grandis* | 31.5 | 0.1701 |
| *Fundulus heteroclitus heteroclitus* | 13.3 | 2.33814 |
| *Fundulus parvipinnis* | 6.34 | 1.31238 |
| *Fundulus similis* | 21 | 0.1029 |
| *Gadus morhua* | 7100 | 957.08 |
| *Gambusia affinis* | 1.1 | 1.63438 |
| *Gambusia holbrooki* | 1.5 | 0.429 |
| *Gasterosteus aculeatus aculeatus* | 2.39 | 0.744246 |
| *Genyagnus monopterygius* | 276 | 5.8512 |
| *Gilchristella aestuaria* | 1.44 | 0.370512 |
| *Gillichthys mirabilis* | 16.7 | 0.09352 |
| *Girella nigricans* | 257 | 22.7702 |
| *Glossogobius giuris* | 23.2 | 2.39192 |
| *Gnathonemus petersii* | 5.85 | 0.364455 |
| *Gobio gobio gobio* | 63.8 | 21.373 |
| *Gobius paganellus* | 10.5 | 1.7745 |
| *Gymnocephalus cernuus* | 64.8 | 12.01392 |
| *Haplochromis elegans* | 9.5 | 0.6099 |
| *Hemichromis bimaculatus* | 3 | 0.3831 |
| *Heteropneustes fossilis* | 75 | 4.26 |
| *Hippocampus hippocampus* | 10 | 1.538 |
| *Hoplerythrinus unitaeniatus* | 533 | 25.6373 |
| *Hoplias malabaricus* | 181.6 | 7.77248 |
| *Hybognathus nuchalis* | 1.7 | 2.02334 |
| *Ichthyomyzon fossor* | 3.78 | 0.689472 |
| *Ictalurus punctatus* | 825 | 29.2875 |
| *Julidochromis marlieri* | 3.9 | 1.15479 |
| *Katsuwonus pelamis* | 3834 | 858.816 |
| *Kuhlia sandvicensis* | 58 | 2.9638 |
| *Labeo calbasu* | 0.3 | 0.00927 |
| *Labeo capensis* | 412.46 | 66.19983 |
| *Labeo rohita* | 11.5 | 3.12685 |
| *Labeobarbus aeneus* | 325.5 | 14.9079 |
| *Labrus bergylta* | 125 | 12.65 |
| *Lagodon rhomboides* | 44 | 32.692 |
| *Lampetra fluviatilis* | 63.6 | 6.37272 |
| *Lampetra planeri* | 2.79 | 0.188883 |
| *Leiostomus xanthurus* | 133.75 | 62.31413 |
| *Lepidocephalichthys guntea* | 1.27 | 0.127508 |
| *Lepidogalaxias salamandroides* | 1.24 | 0.09052 |
| *Lepomis cyanellus* | 10 | 2.052 |
| *Lepomis gibbosus* | 44.92 | 2.0214 |
| *Lepomis macrochirus* | 133.5 | 0.60075 |
| *Leporinus fasciatus* | 7.6 | 0.5358 |
| *Leucaspius delineatus* | 1.52 | 0.6688 |
| *Leuciscus idus* | 900 | 126.9 |
| *Leuciscus leuciscus* | 9.95 | 4.89938 |
| *Limanda limanda* | 400 | 31.52 |
| *Lipolagus ochotensis* | 3.4 | 1.10024 |
| *Lipophrys pholis* | 38 | 12.521 |
| *Liza aurata* | 170 | 67.49 |
| *Liza dumerili* | 79.6 | 4.95112 |
| *Liza macrolepis* | 92 | 14.8028 |
| *Liza richardsonii* | 54.9 | 11.69919 |
| *Lota lota* | 213 | 36.5508 |
| *Lutjanus campechanus* | 450 | 77.355 |
| *Macrognathus aculeatus* | 83.5 | 3.9078 |
| *Melamphaes acanthomus* | 22.7 | 3.32328 |
| *Melanochromis auratus* | 16.4 | 1.06272 |
| *Melanogrammus aeglefinus* | 156 | 25.2408 |
| *Melanonus zugmayeri* | 31.5 | 4.0824 |
| *Melanostigma pammelas* | 10 | 0.944 |
| *Micropterus salmoides* | 350 | 22.19 |
| *Microstomus kitt* | 249 | 22.1112 |
| *Misgurnus fossilis* | 28.5 | 4.1781 |
| *Monopterus cuchia* | 172.88 | 3.267432 |
| *Morone americana* | 264.99 | 68.49992 |
| *Morone saxatilis* | 220.5 | 74.8377 |
| *Mugil cephalus* | 259 | 30.4843 |
| *Mugil curema* | 140 | 13.972 |
| *Myoxocephalus octodecemspinosus* | 341 | 39.2491 |
| *Myoxocephalus scorpius* | 152 | 45.0528 |
| *Mystus armatus* | 9.2 | 0.63204 |
| *Mystus cavasius* | 56 | 6.5968 |
| *Mystus gulio* | 12 | 0.6192 |
| *Mystus vittatus* | 28 | 3.0828 |
| *Myxine glutinosa* | 38.4 | 7.29984 |
| *Nannobrachium regale* | 2.9 | 0.24302 |
| *Nannobrachium ritteri* | 2.1 | 0.47733 |
| *Naucrates ductor* | 0.24 | 1.122624 |
| *Neochanna burrowsius* | 7.1 | 0.48564 |
| *Neolamprologus brichardi* | 5.5 | 1.06755 |
| *Nimbochromis venustus* | 4.2 | 0.71442 |
| *Notemigonus crysoleucas* | 3.6 | 2.88468 |
| *Notothenia cyanobrancha* | 200 | 127.34 |
| *Notothenia rossii* | 400 | 213.4 |
| *Oligolepis acutipennis* | 6.3 | 0.11781 |
| *Oncorhynchus kisutch* | 20.45 | 21.24755 |
| *Oncorhynchus mykiss* | 1200 | 155.28 |
| *Oncorhynchus nerka* | 1432 | 99.524 |
| *Oncorhynchus tshawytscha* | 26 | 6.4116 |
| *Oneirodes acanthias* | 4.2 | 0.25536 |
| *Ophiodon elongatus* | 10000 | 645 |
| *Opsanus tau* | 325 | 130 |
| *Oreochromis aureus* | 697 | 27.8103 |
| *Oreochromis mossambicus* | 150 | 8.115 |
| *Oreochromis niloticus niloticus* | 310 | 10.757 |
| *Orthodon microlepidotus* | 678 | 82.8516 |
| *Oryzias latipes* | 0.42 | 0.177534 |
| *Osphronemus goramy* | 12.5 | 0.57625 |
| *Paranotothenia magellanica* | 200 | 95.58 |
| *Parophrys vetulus* | 70 | 11.046 |
| *Parvilux ingens* | 9.4 | 0.85728 |
| *Perca flavescens* | 7.25 | 2.2243 |
| *Perca fluviatilis* | 201 | 34.3107 |
| *Percina caprodes* | 4.7 | 2.80073 |
| *Petromyzon marinus* | 75.6 | 9.3744 |
| *Pimephales promelas* | 2 | 0.3598 |
| *Pimephales vigilax* | 4.9 | 3.19039 |
| *Platichthys flesus* | 494 | 25.5892 |
| *Platichthys stellatus* | 1475 | 105.4625 |
| *Plecoglossus altivelis altivelis* | 10.7 | 9.34859 |
| *Pleuronectes platessa* | 632 | 78.5576 |
| *Poecilia latipinna* | 5.6 | 0.12488 |
| *Poecilia reticulata* | 1.14 | 0.22344 |
| *Pollachius pollachius* | 870 | 238.032 |
| *Pomadasys commersonnii* | 2627 | 184.1527 |
| *Pomatomus saltatrix* | 225 | 101.565 |
| *Pomoxis annularis* | 11.5 | 1.31675 |
| *Poromitra crassiceps* | 17.1 | 1.43298 |
| *Protopterus annectens annectens* | 368 | 12.9168 |
| *Psetta maxima* | 1000 | 120 |
| *Pseudobathylagus milleri* | 41.1 | 3.44418 |
| *Pseudopleuronectes americanus* | 716 | 65.0844 |
| *Pterophyllum scalare* | 19.03 | 0.985754 |
| *Puntius ticto* | 5.3 | 0.26659 |
| *Pygocentrus nattereri* | 158 | 0.3476 |
| *Rhacochilus vacca* | 3.8 | 1.00548 |
| *Rhinogobiops nicholsii* | 4.08 | 0.405552 |
| *Rhinomugil corsula* | 44.6 | 3.87574 |
| *Rhodeus amarus* | 0.71 | 0.197451 |
| *Rhodeus sericeus* | 3 | 1.1799 |
| *Rhomboplites aurorubens* | 398 | 150.7624 |
| *Rutilus rutilus* | 222 | 25.2858 |
| *Sagamichthys abei* | 5.7 | 0.69654 |
| *Salmo salar* | 227 | 39.7704 |
| *Salmo trutta fario* | 350 | 53.97 |
| *Salmo trutta trutta* | 615 | 92.373 |
| *Salvelinus alpinus alpinus* | 210 | 26.523 |
| *Salvelinus fontinalis* | 1760 | 296.032 |
| *Salvelinus namaycush* | 82.8 | 5.60556 |
| *Sander lucioperca* | 650 | 391.105 |
| *Sander vitreus* | 674 | 49.539 |
| *Sarda chiliensis lineolata* | 2530 | 3684.439 |
| *Sarotherodon galilaeus galilaeus* | 283 | 11.9709 |
| *Scardinius erythrophthalmus* | 53 | 11.501 |
| *Scopelengys tristis* | 49.8 | 3.42624 |
| *Scopelogadus mizolepis mizolepis* | 3.6 | 0.072 |
| *Scophthalmus rhombus* | 145 | 21.489 |
| *Scorpaena porcus* | 50 | 3.285 |
| *Scyliorhinus canicula* | 857 | 72.9307 |
| *Scyliorhinus stellaris* | 2800 | 14.28 |
| *Sebastes diploproa* | 2 | 0.4508 |
| *Sebastolobus altivelis* | 198 | 3.0294 |
| *Seriola quinqueradiata* | 989 | 156.8554 |
| *Serranus scriba* | 4.1 | 0.9758 |
| *Solea solea* | 185 | 33.855 |
| *Sparus aurata* | 78.3 | 17.33562 |
| *Spinachia spinachia* | 5 | 2.018 |
| *Squalius cephalus* | 168 | 36.0528 |
| *Squalus acanthias* | 8970 | 432.354 |
| *Steatocranus tinanti* | 4 | 0.3132 |
| *Stenobrachius leucopsarus* | 4.4 | 1.18624 |
| *Stomias atriventer* | 9.3 | 1.4322 |
| *Symbolophorus californiensis* | 0.8 | 0.36576 |
| *Synbranchus marmoratus* | 199 | 5.0347 |
| *Syngnathus acus* | 7.6 | 1.68112 |
| *Tarletonbeania crenularis* | 3.29 | 1.559131 |
| *Tautogolabrus adspersus* | 50 | 7.1 |
| *Theragra chalcogramma* | 300 | 82.86 |
| *Thorichthys meeki* | 15 | 0.8085 |
| *Thunnus alalunga* | 13600 | 5877.92 |
| *Thymallus arcticus arcticus* | 283 | 73.4102 |
| *Tilapia rendalli* | 50 | 5.445 |
| *Tilapia zillii* | 315 | 6.048 |
| *Tinca tinca* | 541 | 33.2174 |
| *Torpedo marmorata* | 3300 | 133.32 |
| *Torpedo torpedo* | 410 | 49.815 |
| *Trichogaster trichopterus* | 7.97 | 0.690202 |
| *Triphoturus mexicanus* | 9.3 | 2.3994 |
| *Tropheus moorii* | 10.9 | 2.55169 |
| *Typhlogobius californiensis* | 3.54 | 0.011328 |
| *Xiphophorus hellerii* | 2 | 0.2832 |
| *Zoarces viviparus* | 0.29 | 0.111389 |
