# Supplementary material for: Global Human Footprint on the Linkage between Biodiversity and Ecosystem Functioning in Reef Fishes
Source: PLoS Biol. 2011 Apr 5;9(4):e1000606. doi: 10.1371/journal.pbio.1000606 (PMC3071368; doi:10.1371/journal.pbio.1000606)
Supplement: Table S5 — Data used to establish the link between biomass production and body mass in fishes. (0.10 MB DOC) [file pbio.1000606.s010.doc]

Table S5. Data used to establish the link between biomass production and body mass in fishes [see Fig. S1g]. Data were gathered as part of the literature reviews carried out by Graham Edgar [Edgard & Shaw 1995] and Morgan Ernest [Ernest et al. 2003]. The former is a coauthor of this paper while the latter kindly provided her data for this analysis.

| **Species** | **Weight**  **[g]** | **Production**  **[g/year]** | **Source** |
| --- | --- | --- | --- |
| *Alburnus alburnus* | 6.9200 | 11.1135 | Banse & Mosher [1980] |
| *Aldrichetta forsteri* | 5.8200 | 41.6100 | Chubb et al. [1991] |
| *Archosargus probatocephalus* | 221.0000 | 206.9550 | Schwartz [1990] |
| *Argyrozona argyrozona* | 98.8000 | 65.3350 | Nepgen [1977] |
| *Atherinosoma presbyteroides* | 0.4070 | 1.2739 | Prince & Potter [1983] |
| *Caranx georgianus* | 1750.0000 | 1591.4000 | James [1984] |
| *Carcharinus tilstoni* | 3930.0000 | 2219.2000 | Davenport & Stevens [1988] |
| *Chanos chanos* | 7.3400 | 121.1800 | Kumagai et al. [1985] |
| *Cheilodactylus macropterus* | 319.0000 | 132.4950 | Tong & Vooren [1972] |
| *Chelidonichthys kumu* | 76.0000 | 58.0350 | Elder [1976] |
| *Chrysophrys auratus* | 1320.0000 | 996.4500 | Vooren & Coombs [1977] |
| *Clupea harengus* | 1.6500 | 18.1040 | De Silva [1973] |
| *Clupea pallasii* | 52.1000 | 53.2900 | Kanno [1989] |
| *Coracinus capensis* | 215.0000 | 162.4250 | Bennett & Griffiths [1986] |
| *Coryphaena hippurus* | 12300.0000 | 23287.0000 | Oxenford & Hunte [1981] |
| *Cymatoceps nasutus* | 276.0000 | 136.8750 | Buxton & Clarke [1989] |
| *Cynoscion arenarius* | 74.4000 | 302.5850 | Shlossman & Chittenden [1981] |
| *Cynoscion nothus* | 24.1000 | 169.7250 | DeVries & Chittenden [1982] |
| *Cynoscion regalis* | 0.1190 | 3.5077 | Szedmayer et al. [1990] |
| *Engraulis capensis* | 0.0952 | 2.6025 | Thomas [1986] |
| *Engraulis mordax* | 5.6100 | 9.3075 | Parrish et al. [1985] |
| *Etheostoma flabellare* | 1.2300 | 2.3794 | Banse & Mosher [1980] |
| *Etheostoma spectabile* | 0.5380 | 1.3942 | Banse & Mosher [1980] |
| *Etrumeus micropus* | 14.2000 | 23.6885 | Geldenhuys [1978] |
| *Fundulus heteroclitus* | 0.6440 | 1.6097 | Kneib & Stiven [1978] |
| *Gadus morhua* | 15000.0000 | 2190.0000 | Hutchings [1999] |
| *Galeorhinus australis* | 4040.0000 | 1620.6000 | Grant et al. [1979] |
| *Gobio gobio* | 9.2300 | 18.1923 | Banse & Mosher [1980] |
| *Gobius cobitis* | 1.6700 | 2.8616 | Gibson [1970] |
| *Gobius paganellus* | 0.4930 | 3.3434 | Miller [1961] |
| *Helicolenus lengerichi* | 36.7000 | 41.2450 | Petrova & Chekunova [1979] |
| *Helicolenus percoides* | 15.0000 | 18.2135 | Withell & Wankowski [1988] |
| *Hippoglossoides dubius* | 4.7600 | 6.6795 | Nakatani et al.[1990] |
| *Hippoglossoides platessoides* | 277.0000 | 111.2155 | Banse & Mosher [1980] |
| *Katsuwonus pelamis* | 1860.0000 | 3420.0500 | Uchiyama & Struhsaker [1981] |
| *Lagodon rhomboides* | 0.6760 | 2.7375 | Hansen [1969] |
| *Lates calcarifer* | 3070.0000 | 1408.9000 | Davis & Kirkwood [1984] |
| *Leionura atun* | 40.4000 | 176.2950 | Grant et al. [1978] |
| *Leiostomus xanthurus* | 0.0013 | 0.0314 | Warlen & Chester [1985] |
| *Lesueurigobius friesii* | 0.2030 | 0.9162 | Gibson & Ezzi [1978] |
| *Leuciscus leuciscus* | 13.8000 | 24.1776 | Banse & Mosher [1980] |
| *Liza subviridis* | 65.6000 | 57.6700 | Al-Daham & Wahab [1991] |
| *Lutjanus campechanus* | 34.4000 | 197.4650 | Holt & Arnold [1982] |
| *Lutjanus kasmira* | 110.0000 | 75.5550 | Morales-Nin & Ralston [1990] |
| *Menidia menidia* | 3.0000 | 13.8700 | Conover & Ross [1982] |
| *Micropogon undulatus* | 0.0714 | 1.9929 | Knudsen & Herke [1978] |
| *Mugil cephalus* | 72.0000 | 199.6550 | Grant & Spain [1975] |
| *Mugil curema* | 9.7800 | 138.3350 | Richards & Castagna [1976] |
| *Mustelus munazo* | 591.0000 | 325.5800 | Tanaka & Mizue [1979] |
| *Nematolosa vlaminghi* | 63.4000 | 41.2450 | Chubb & Potter [1986] |
| *Nemipterus peronii* | 83.4000 | 99.2800 | Sainsbury & Whitelaw [1984] |
| *Oligocottus maculosus* | 1.3000 | 1.2994 | Pierce & Pierson [1990] |
| *Pachymetopon blochii* | 11.8000 | 16.8995 | Pulfrich & Griffiths [1988] |
| *Pagrus pagrus* | 145.0000 | 159.1400 | Manooch & Huntsman [1977] |
| *Pimephales promelas* | 1.7700 | 4.9746 | Banse & Mosher [1980] |
| *Pomatoschistus microps* | 0.5000 | 1.7155 | Healey [1972] |
| *Pseudopleuronectes americanus* | 0.4014 | 3.9420 | Mulkana [1966] |
| *Rhombosolea plebeia* | 3.4700 | 32.8865 | Colman [1978] |
| *Rutilus rutilus* | 15.4000 | 14.0525 | Banse & Mosher [1980] |
| *Sardinops neopilchardus* | 3.6600 | 14.0160 | Blackburn [1949] |
| *Sardinops ocellata* | 0.8560 | 12.2640 | Thomas [1985] |
| *Scomberomorus cavalla* | 0.0051 | 0.6023 | DeVries et al. [1990] |
| *Sebastes serranoides* | 131.0000 | 94.9000 | Love & Westphal [1981] |
| *Sprattus sprattus* | 1.6500 | 18.1040 | De Silva [1973] |
| *Squalus acanthias* | 235.0000 | 90.5200 | Ketchen [1975] |
| *Stolephorus purpureus* | 0.2770 | 2.9346 | Struhsaker & Uchiyama [1976] |
| *Tanakius kitaharai* | 3.4700 | 6.2050 | Yabuki [1989] |
| *Tautolabrus adspersus* | 9370.0000 | 18396.0000 | Dew [1976] |
| *Trachurus declivus* | 243.0000 | 168.2650 | Webb & Grant [1979] |
| *Trachurus trachurus* | 21.0000 | 31.7915 | Geldenhuys [1973] |
| *Trisopterus esmarkii* | 10.5000 | 40.1500 | Gordon [1977] |
