# Supplementary material for: Global Human Footprint on the Linkage between Biodiversity and Ecosystem Functioning in Reef Fishes
Source: PLoS Biol. 2011 Apr 5;9(4):e1000606. doi: 10.1371/journal.pbio.1000606 (PMC3071368; doi:10.1371/journal.pbio.1000606)
Supplement: Text S1 — Sources of data. (0.07 MB DOC) [file pbio.1000606.s011.doc]

**Sources**

Al-Daham, N.K. and Wahab, N.K. Age, growth and reproduction of the greenback mullet, *Liza subviridis* [Valenciennes], in an estuary in southern Iraq. *J. Fish Biol.* **38**, 81–88 [1991].

Arbuckle, J.L. AMOS^TM^ 18 User’s Guide (AMOS Development Corporation, Chicago) [2009].

Banse, K., and Mosher, S. Adult body mass and annual production/biomass relationships of field populations. *Ecol. Monogr.* **50**, 355-379 [1980].

Bellwood, D.R., Hughes, T.P., Folke, C. & Nystrom, N. Confronting the coral reef crisis. *Nature* **429**, 827-833 [2004].

Bennett, B.A. and Griffiths, C.L. Aspects of the biology of galjoen *Coracinus capensis* [Cuvier] off the South-Western Cape, South Africa. *S. Afr. J. Mar. Sci.* **4**, 153–162 [1986].

Berumen, M.L. & Pratchett, M.S. Trade-offs associated with dietary specialization in corallivorous butterflyfishes [Chaetodontidae: Chaetodon]. Behav Ecol Sociobiol 62, 989-994 [2008].

Blackburn, B. Age, rate of growth, and general life-history of the Australian pilchard [*Sardinops neopilchardus*] in New South Wales. *CSIRO Bull.* **242**, 1–86 [1949].

Brown, J.H. et al. Toward a metabolic theory of ecology. *Ecology* 85, 1771-1789 [2004].

Buxton, C.D. and Clarke, J.R. The growth of *Cymatoceps nasutus* [Teleostei: Sparidae], with comments on diet and reproduction. *S. Afr. J. Mar. Sci.* **8**, 57–65 [1989].

Caley, J. & Munday, P.L. Growth trades off with habitat specialization. Proc R Soc Lond B **S270**, 175-177

Cardinale B.J. Effects of biodiversity on the functioning of trophic groups and ecosystems. *Nature* **443**, 989-992 [2006].

Chubb, C.F., Potter, I.C., Grant, C.J., Lenanton, R.C.J. and Wallace, J. Age structure, growth rates and movements of sea mullet, *Mugil cephalus* L., and yellow-eye mullet, *Aldrichetta forsteri* [Valenciennes], in the Swan-Avon River system, Western Australia. *Aust. J. Mar. Freshwater Res.* **32**, 605–628 [1981].

Clarke, A., Johnston, N. M. Scaling of metabolic rate with body mass and temperature in teleost fish. *J. An. Ecol.* **68**, 893-905 [1999].

Colman, J.A. Tagging experiments on the sand flounder, *Rhombosolea plebia* [Richardson], in Canterbury, New Zealand, 1964 to 1966. *Fish. Res. Bull., Fish. Res. Div. N.Z.* **18**, 1–42 [1978].

Conover D.O. & Munch, S.B. Sustaining fisheries yields over evolutionary time scales. Science **297**, 94-96 [2002].

Conover, D.O. and Ross, M.R. Patterns in seasonal abundance, growth and biomass of the Atlantic silverside, *Menidia menidia*, in a New England estuary. *Estuaries* **5**, 275–286 [1982].

Davenport, S. and Stevens, J.D. Age and growth of two commercially important sharks [*Carcharinus tilstoni* and *C. sorrah*] from northern Australia. *Aust. J. Mar. Freshwater Res.* **39**, 413–433 [1988].

Davis, T.L.O. and Kirkwood, G.P. Age and growth studies on barramundi, *Lates calcarifer* [Bloch], in northern Australia. *Aust. J. Mar. Freshwater Res.* **35**, 673–689 [1984].

De Silva, S.S. Abundance, structure, growth and origin of inshore clupeid populations of the west coast of Scotland. *J. Exp. Mar. Biol. Ecol.* **12**, 119–144 [1973].

De Silva, S.S. Abundance, structure, growth and origin of inshore clupeid populations of the west coast of Scotland. *J. Exp. Mar. Biol. Ecol.* **12**, 119–144 [1973].

De Vries, D.A., Grimes, C.B., Lang, K.L. and White, D.B. Age and growth of king and Spanish mackerel larvae and juveniles from the Gulf of Mexico and U.S. South Atlantic Bight. *Environ. Biol. Fish.* **29**, 135–143 [1990].

DeVries, D.A. and Chittenden Jr., M.E. Spawning, age determination, longevity, and mortality of the silver seatrout, *Cynoscion nothus*, in the Gulf of Mexico. *Fish. Bull.* **80**, 487–500 [1982].

Dew, C.B. A contribution to the life history of the cunner, *Tautogolabrus adspersus*, in Fishers Island Sound, Connecticut. *Chesapeake Sci.* **17**, 101–113 [1976].

Diaz, S. and Cabido, MVive la difference: plant functional diversity matters to ecosystem processes. *Trends Ecol. Evol.* **16**, 646–655 [2001].

Edgar, G.J. and Shaw, C. The production and trophic ecology of shallow-water fish assemblages in southern Australia I. Species richness, size-structure and production of fishes in Western Port, Victoria. J. Exp. Mar. Biol. Ecol. **194**, 53-81 [1995].

Elder, R.D. Studies on age and growth, reproduction and population dynamics of red gurnard, *Chelidonichthys kumu* [Lesson and Garnot], in the Hauraki Gulf, New Zealand. *Fish. Res. Bull. Fish. Res. Div. N.Z.* **12**, 1–77 [1976].

Ernest, S.K.M. et al. Thermodynamic and metabolic effects on the scaling of production and population energy use. *Ecol. Lett*. 6, 990-995 [2003].

Geldenhuys, N.D. Age determination of the South African round herring *Etrumeus micropus* and length and age composition of the commercial catches, 1965–1973. *Sea Fish. Br. Invest. Rep. S.A. Dept. Industries* **115**, 1–16 [1978].

Geldenhuys, N.D. Growth of the South African maasbanker *Trachurus trachurus* Linnaeus and age composition of the catches, 1950–1971. *Sea Fish. Br. Invest. Rep. S.A. Dept. Industries* **101**, 1–24 [1973].

Gibson, R.N. and Ezzi, I.A. The biology of a Scottish population of Fries' goby, *Lesueurigobius friesii*. *J. Fish Biol.* **12**, 371–389 [1978].

Gibson, R.N. Observations on the biology of the giant goby *Gobius cobitis* Pallas. *J. Fish Biol.* **2**, 281–288 [1970].

Grant, C.J. and Spain, A.V. Reproduction, growth and size allometry of *Mugil cephalus* Linnaeus [Pisces: Mugilidae] from North Queensland inshore waters. *Aust. J. Zool.* **23**, 181–201 [1975].

Grant, C.J., Cowper, T.R. and Reid, D.D. Age and growth of snoek, *Leionura atun* [Euphrasen], in south-eastern Australian waters. *Aust. J. Mar. Freshwater Res.* **29**, 435–444 [1978].

Grant, C.J., Sandland, R.L. and Olsen, A.M. Estimation of growth, mortality and yield per recruit of the Australian school shark, *Galeorhinus australis* [Macleay], from tag recoveries. *Aust. J. Mar. Freshwater Res.* **30**, 625–637 [1979].

Gross, K., and B. J. Cardinale. 2005. The functional consequences of random verses ordered species extinctions. Ecology Letters 8:409-418.

Hansen, D.J. Food, growth, migration, reproduction, and abundance of pinfish, *Lagodon rhomboides*, and Atlantic croaker, *Micropogon undulatus*, near Pensacola, Florida, 1963–1965. *Fish. Bull.* **68**, 135–146 [1969].

Healey, M.C. On the population ecology of the common goby in the Ythan estuary. *J. Nat. Hist.* **6**, 133–145 [1972].

Hixon, M & Carr, M. Synergistic predation, density dependence, and population regulation in marine fish. Science **277**, 946-949 [1997]

Hixon, M. Predation as a process structuring coral reef fish communities. In Sale PF [ed] the Ecology of fishes on coral reefs. Academic Press, San Diego, 2001]

Holt, S.A. and Arnold, C.R. Growth of juvenile red snapper *Lutjanus campechanus*, in the northwestern Gulf of Mexico. *Fish. Bull.* **80**, 644–648 [1982].

Hutchings, J.A. The influence of growth and survival costs of reproduction on Atlantic cod, *Gadus morhua*, population growth rate. *Can. J. Fish. Aquatic Sci.* **56**, 1612-1623 [1999].

James, G.D. Trevally, *Caranx georgianus* Cuvier: Age determination, population biology, and the fishery. *Fish. Res. Bull. Fish. Res. Div. N.Z.* **25**, 1–51 [1984].

Kanno, Y. Comparison of age composition, sex ratio and growth rate among populations of herring *Clupea pallasii* in the Far Eastern waters. *Nippon Suisan Gakkaishi* **55**, 583–589 [1989].

Ketchen, K.S. Age and growth of dogfish *Squalus acanthias* in British Columbian waters. *J. Fish. Res. Board Can.* **32**, 43–59 [1975].

Kneib, R.T. and Stiven, A.E. Growth, reproduction, and feeding of *Fundulus heteroclitus* [L.] on a North Carolina saltmarsh. *J. Exp. Mar. Biol. Ecol.* **31**, 121–140 [1978].

Knudsen, E.E. and Herke, W.H. Growth rate of marked juvenile Atlantic croakers, *Micropogon undulatus*, and length of stay in a coastal marsh nursery in southwest Louisiana. *Trans. Am. Fish. Soc.* **107**, 12–20 [1978].

Kumagai, S., Bagarinao, T. and Unggui, A. Growth of juvenile milkfish *Chanos chanos* in a natural habitat. *Mar. Ecol. Prog. Ser.* **22**, 1–6 [1985].

Love, M.S. and Westphal, W.V. Growth, reproduction, and food habits of olive rockfish, *Sebastes serranoides*, off central California. *Fish. Bull.* **79**, 533–545 [1981].

Manooch, C.S. and Huntsman, G.R. Age, growth, and mortality of the red porgy, *Pagrus pagrus*. *Trans. Am. Fish. Soc.* **106**, 26–33 [1977].

Miller, P.J. Age, growth and reproduction of the *Gobius paganellus* L., in the Isle of Man. *J. Mar. Biol. Assoc. U.K.* **41**, 737–769 [1961].

Mora, C. et al. Coral reefs and the global network of marine protected areas. *Science* **312**, 1750–1751 [2006].

Morales-Nin, B. and Ralston, S. Age and growth of *Lutjanus kasmira* [Forskal] in Hawaiian waters. *J. Fish Biol.* **36**, 191–203 [1990].

Mulkana, M.S. The growth and feeding habits of juvenile fishes in two Rhode Island estuaries. *Gulf Res. Rep.* **2**, 97–167 [1966].

Nakatani, T., Koizumi, H., Yokoyama, S., Maeda, T., Takahashi, T. and Matsushima, H. Age and growth of pleuronectid flounder *Hippoglossoides dubius* in Funka Bay, Hokkaido. *Nippon Suisan Gakkaishi* **56**, 893–901 [1990].

Nepgen, C.S.de V. The biology of the hottentot *Pachymetopon blochii* [Val.] and the silverfish *Argyrozona argyrozona* [Val.] along the Cape south-west coast. *Sea Fish. Br. Invest. Rep. S.A. Dept. Industries* **105**, 1–35 [1977].

Oxenford, H.A. and Hunte, W. Age and growth of dolphin, *Coryphaena hippurus*, as determined by growth rings in otoliths. *Fish. Bull.* **81**, 906–909 [1981].

Parrish, R.H., Mallicoate, D.L. and Mais, K.F. Regional variations in the growth and age composition of northern anchovy, *Engraulis mordax*. *Fish. Bull.* **83**, 483–496 [1985].

Pauly, D. On the interrelationships between natural mortality, growth parameters, and mean environmental temperature in 175 fish stocks. *Journal du Conseil* **39**, 175– 192 [1980].

Peters, R.H. *The Ecological Implications of Body Size*. Cambridge Press [1986].

Petrova, Y.G. and Chekunova, V.I. Growth of the Chilean perch. *Hydrobiol. J.* **15**, 41–46 [1979].

Pierce, B.E. and Pierson, K.B. Growth and reproduction of the tidepool sculpin *Oligocottus maculosus*. *Jpn. J. Ichthyol.* **36**, 410–418 [1990].

Prince, J.D. and Potter, I.C. Life-cycle duration, growth and spawning times of five species of Atherinidae [Teleostei] found in a Western Australian estuary. *Aust. J. Mar. Freshwater Res.* **34**, 287–301 [1983].

Pulfrich, A. and Griffiths, C.L. Growth, sexual maturity and reproduction in the hottentot *Pachymetopon blochii* [Val.]. *S. Afr. J. Mar. Sci.***7**, 25–36 [1988].

Richards, C.E. and Castagna, M. Distribution, growth, and predation of juvenile white mullet [*Mugil curema*] in oceanside waters of Virginia's eastern shore. *Chesapeake Science* **17**, 308–309 [1976].

Sainsbury, K.J. and Whitelaw, A.W. Biology of Peron's threadfin bream, *Nemipterus peroni* [Valenciennes], from the North West Shelf of Australia. *Aust. J. Mar. Freshwater Res.* **35**, 167–185 [1984].

Savage, V.M. et al. Effects of body size and temperature on population Growth. Amer Nat **163**, 429-441 [2004]

Savage, V.M. et al. The predominance of quarter-power scaling in biology. *Functional Ecology* **18**, 257-282, [2004].

Schermelleh-Engel. K. and Moosbrugger, H. Evaluating the fit of structural equation models: tests of significance and descriptive goodness-of-fit measures. Methods of Psychological Research 8, 23-74 [2003].

Schwartz, F.J. Length-weight, age and growth, and landings observations for sheepshead *Archosargus probatocephalus* from North Carolina. *Fish. Bull.* **88**, 829–832 [1990].

Shlossman, P.A. and Chittenden Jr., M.E.. Reproduction, movements, and population dynamics of the sand seatrout, *Cynoscion arenarius*. *Fish. Bull.* **79**, 649–669 [1981].

Sogard, S. Size-Selective Mortality in the Juvenile Stage of Teleost Fishes: A Review. Bull Mar Sci 60, 1129-1157 [1997]

Struhsaker, P. and Uchiyama, J.H. Age and growth of the nehu, *Stolephorus purpureus* [Pisces: Engraulidae], from the Hawaiian Islands as indicated by daily growth increments of sagittae. *Fish. Bull.* **74**, 9–17 [1976].

Szedmayer, S.T., Weinstein, M.P. and Musick, J.A. Differential growth among cohorts of Age-0 weakfish *Cynoscion regalis* in Chesapeake Bay. *Fish. Bull.* **88**, 745–752 [1990].

Tanaka, S. and Mizue, K. Studies on sharks-XV Age and growth of the Japanese dogfish *Mustelus manazo* Bleeker in the East China Sea. *Bull. Jpn. Soc. Sci. Fish.* **45**, 43–50 [1979].

Thomas, R.M. Growth of larval pelagic fish in the south-east Atlantic from daily otolith rings in 1982/83 and 1983/84. *S. Afr. J. Mar. Sci.* **4**, 61–77 [1986].

Thomas, R.M. Growth rate of the pilchard off south west Africa, 1971–1983. *Sea Fish. Res. Inst. Invest. Rep. S.A. Dept. Environ. Affairs* **128**, 1–41 [1985].

Tong, L.J. and Vooren, C.M. The biology of the New Zealand Tarakihi, *Cheilodactylus macropterus* [Bloch and Schneider]. *Fish. Res. Bull. Fish. Res. Div. N.Z.* **6**, 1–60 [1972].

Uchiyama, J.H. and Struhsaker, P. Age and growth of skipjack tuna, *Katsuwonus pelamis*, and yellowfin tuna, *Thunnus albacares*, as indicated by daily growth increments of sagittae. *Fish. Bull.* **79**, 151–162 [1981].

Vooren, C.M. and Coombs, R.F. Variations in growth, mortality, and population density of snapper, *Chrysophrys auratus* [Forster], in the Hauraki Gulf, New Zealand. *Fish. Res. Bull. Fish. Res. Div. N.Z.* **14**, 1–32 [1977].

Warlen, S.M. and Chester, A.J. Age, growth, and distribution of larval spot, *Leiostomus xanthurus*, off North Carolina. *Fish. Bull.* **83**, 587–600 [1985].

Webb, B.F. and Grant, C.J. Age and growth of jack mackerel, *Trachurus declivis* [Jenyns], from south-eastern Australian waters. *Aust. J. Mar. Freshwater Res.* **30**, 1–9 [1979].

Withell, A.F. and Wankowski, J.W. Estimates of age and growth of ocean perch, *Helicolenus percoides* Richardson, in south-eastern Australian waters. *Aust. J. Mar. Freshwater Res.* **39**, 441–457 [1988].

Wright, J.P. et al. Conventional functional classification schemes underestimate the relationship with ecosystem functioning. *Ecol. Lett.* **9**, 111-120 [2006].

Yabuki, K. Age determination of yanagimushigarei *Tanakius kitaharai* [Pleuronectidae] from otoliths in the Sea of Japan off Kyoto Prefecture. *Nippon Suisan Gakkaishi* **55**, 1331–1338 [1989].
